# Supplementary figures and images for: Nuclear and cytosolic J-domain proteins provide synergistic control of Hsf1 at distinct phases of the heat shock response
Source: eLife. 2025 Sep 30;14:RP107157. doi: 10.7554/eLife.107157 (PMC12483511; doi:10.7554/eLife.107157)

A

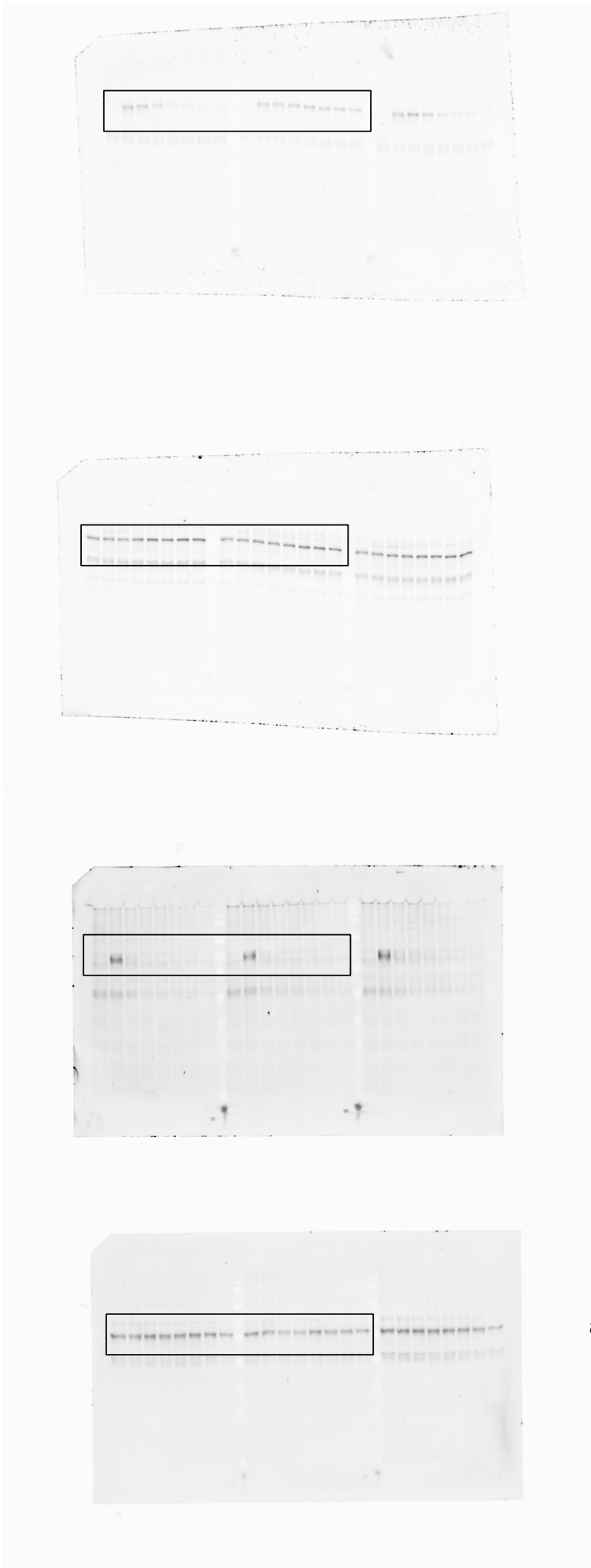

anti-Btn2

anti-Zwf1

B

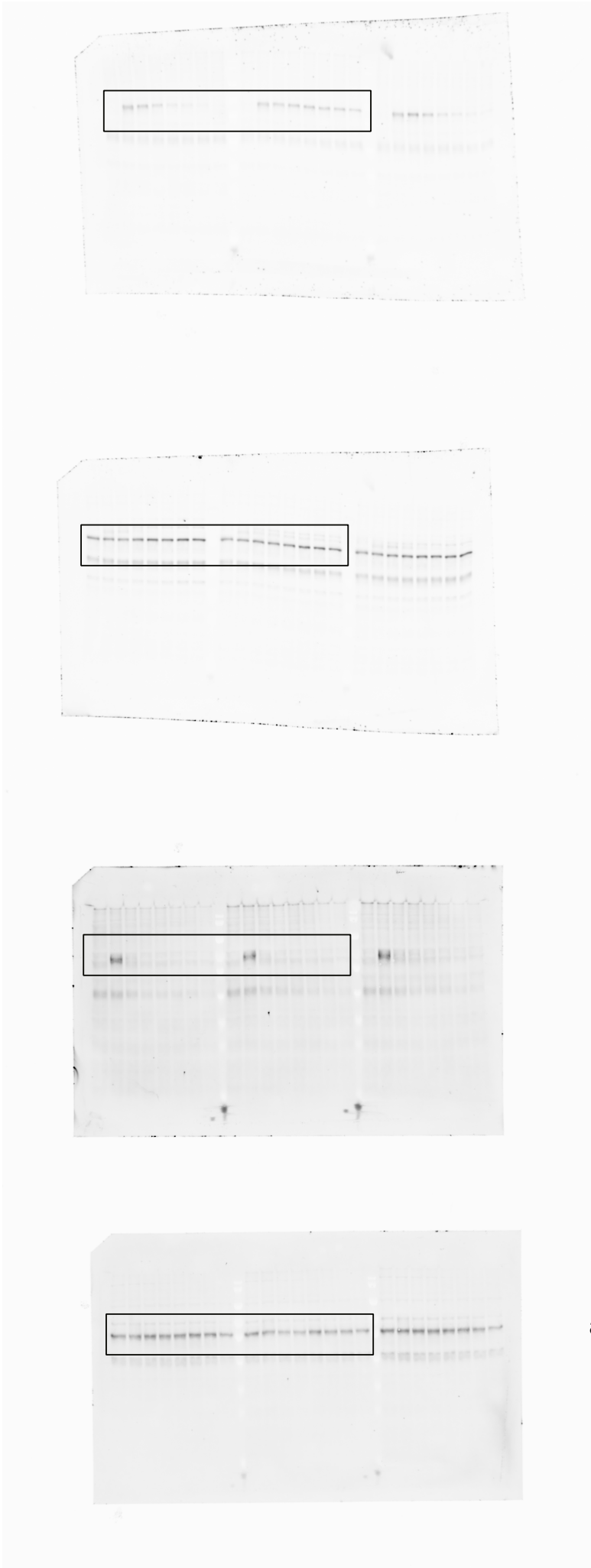

anti-Btn2

anti-Zwf1

Supplement: Figure 1—source data 1. [file elife-107157-fig1-data1.zip › Figure 1A:B-source data 1/Source File Figure1AB.pdf]

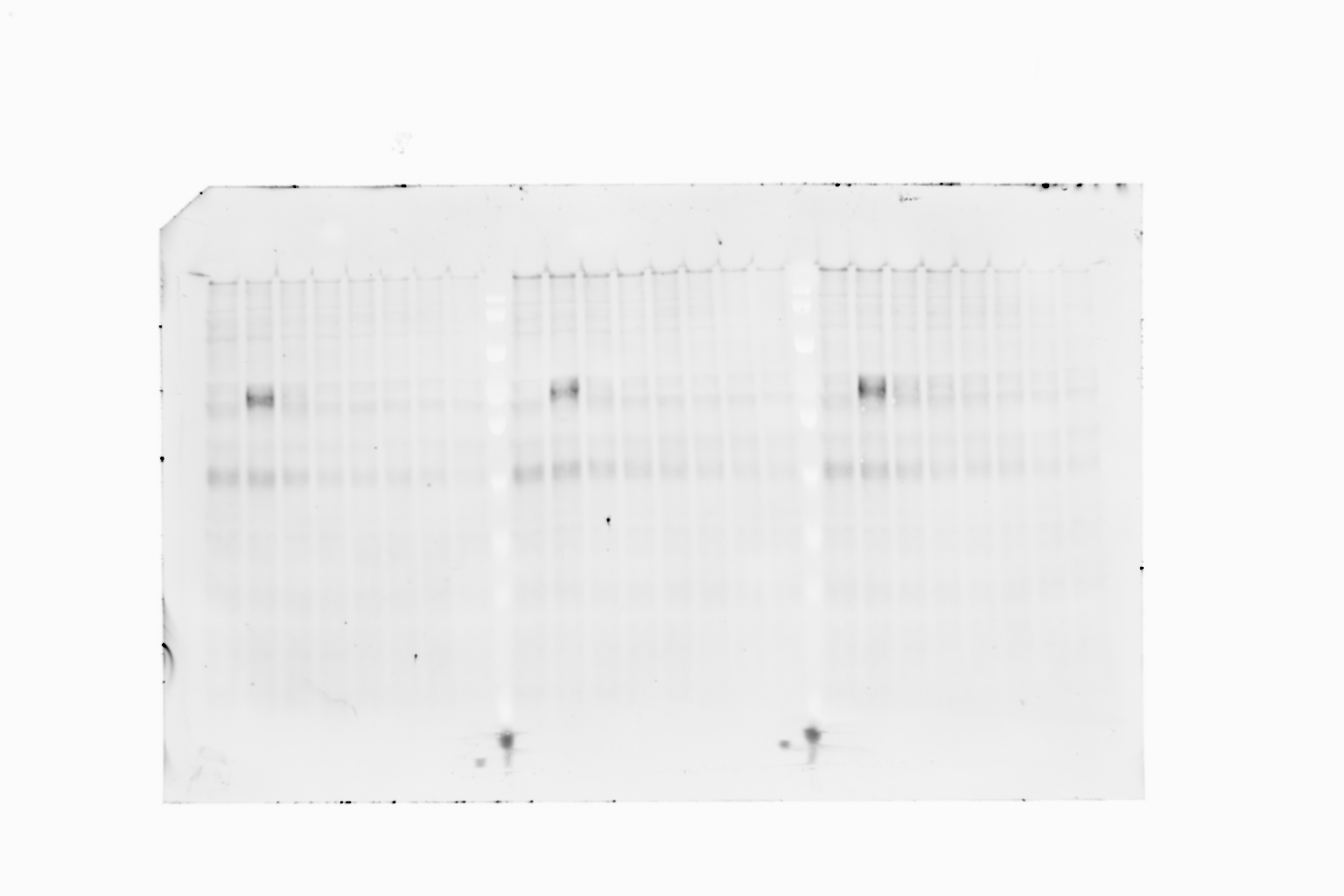

Supplement: Figure 1—source data 2. [file elife-107157-fig1-data2.zip › Figure 1A:B-source data 2/Figure 1B_anti Btn2.tif]

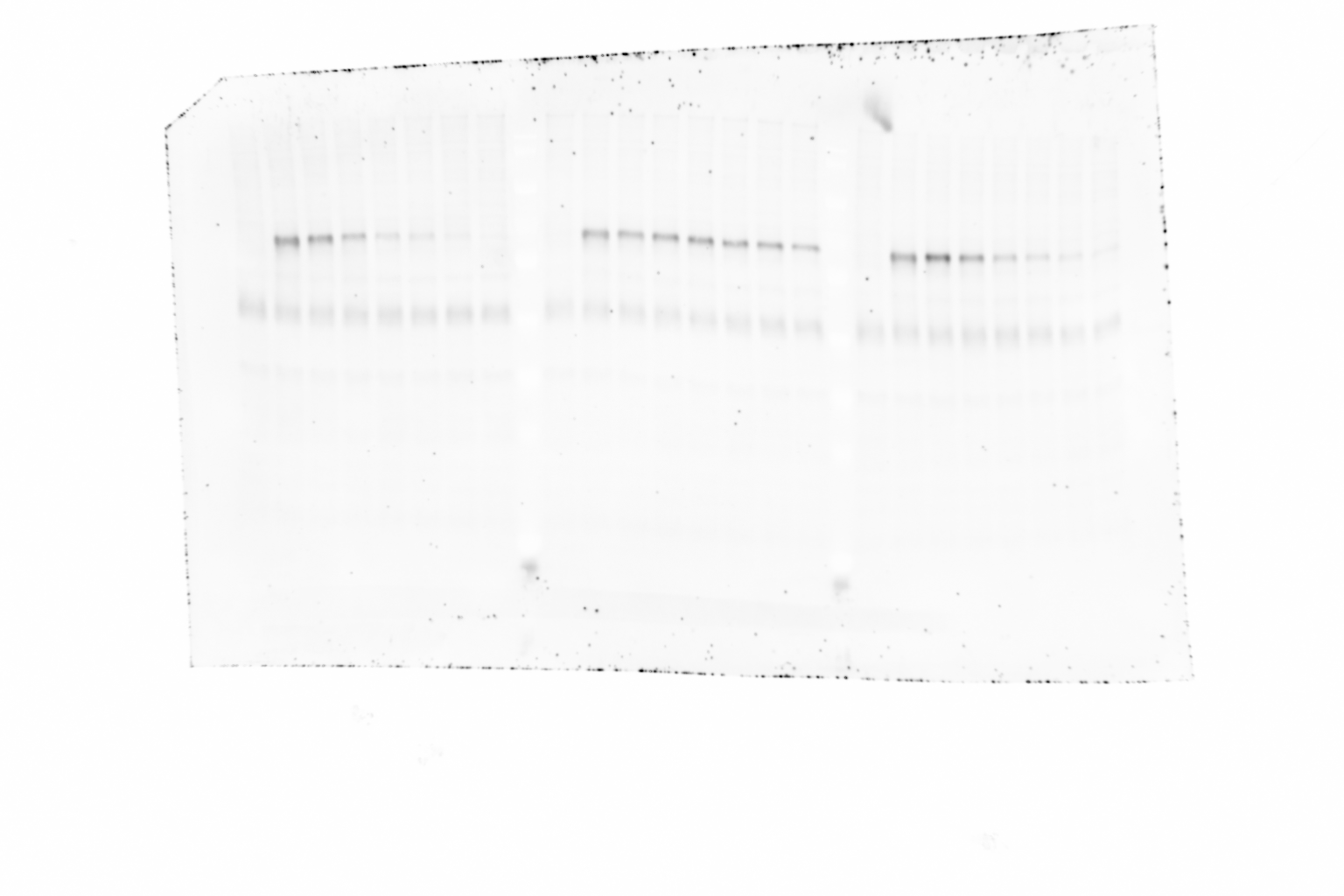

Supplement: Figure 1—source data 2. [file elife-107157-fig1-data2.zip › Figure 1A:B-source data 2/Figure 1A_anti Btn2 .tif]

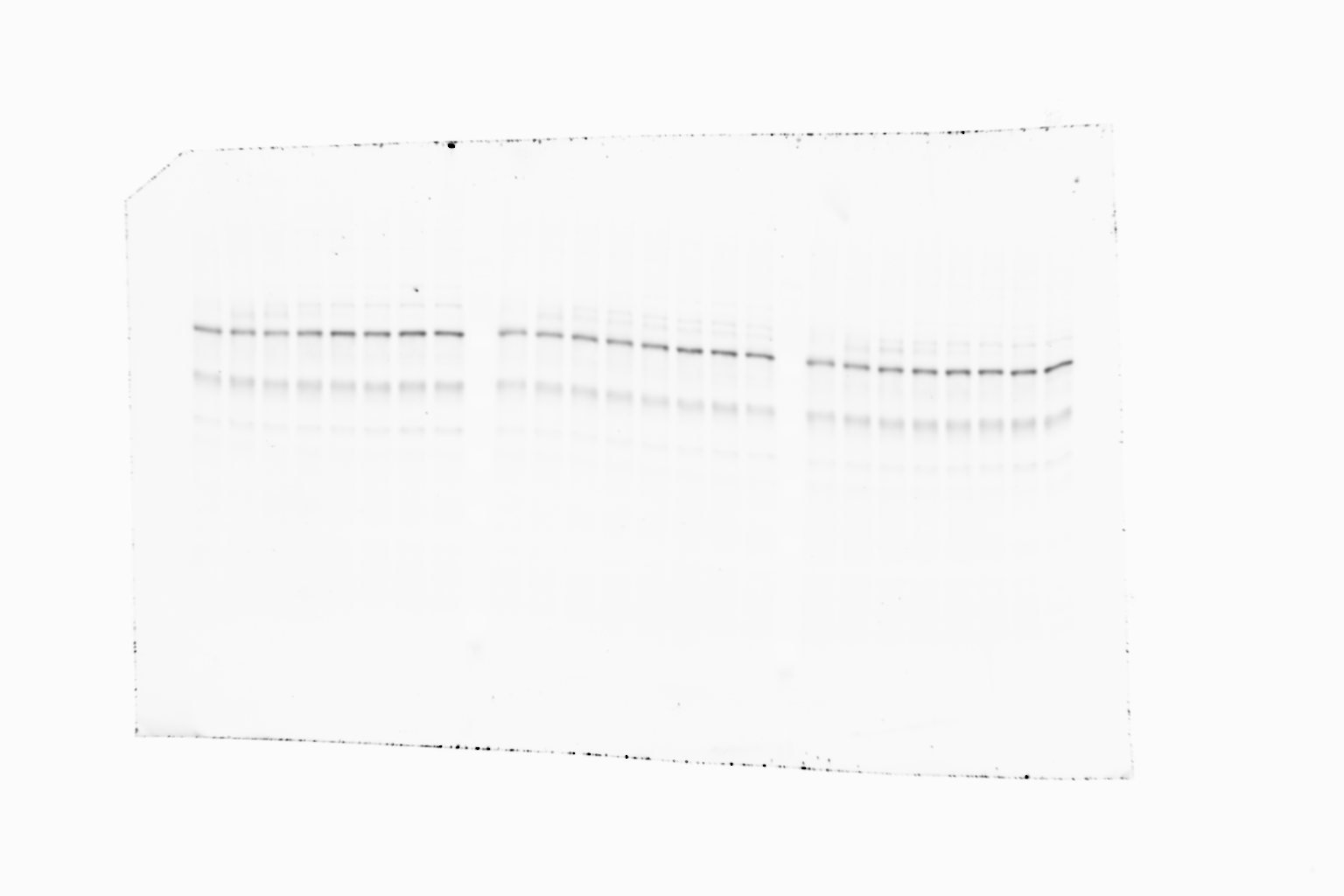

Supplement: Figure 1—source data 2. [file elife-107157-fig1-data2.zip › Figure 1A:B-source data 2/Figure1A_anti Zwf1.tif]

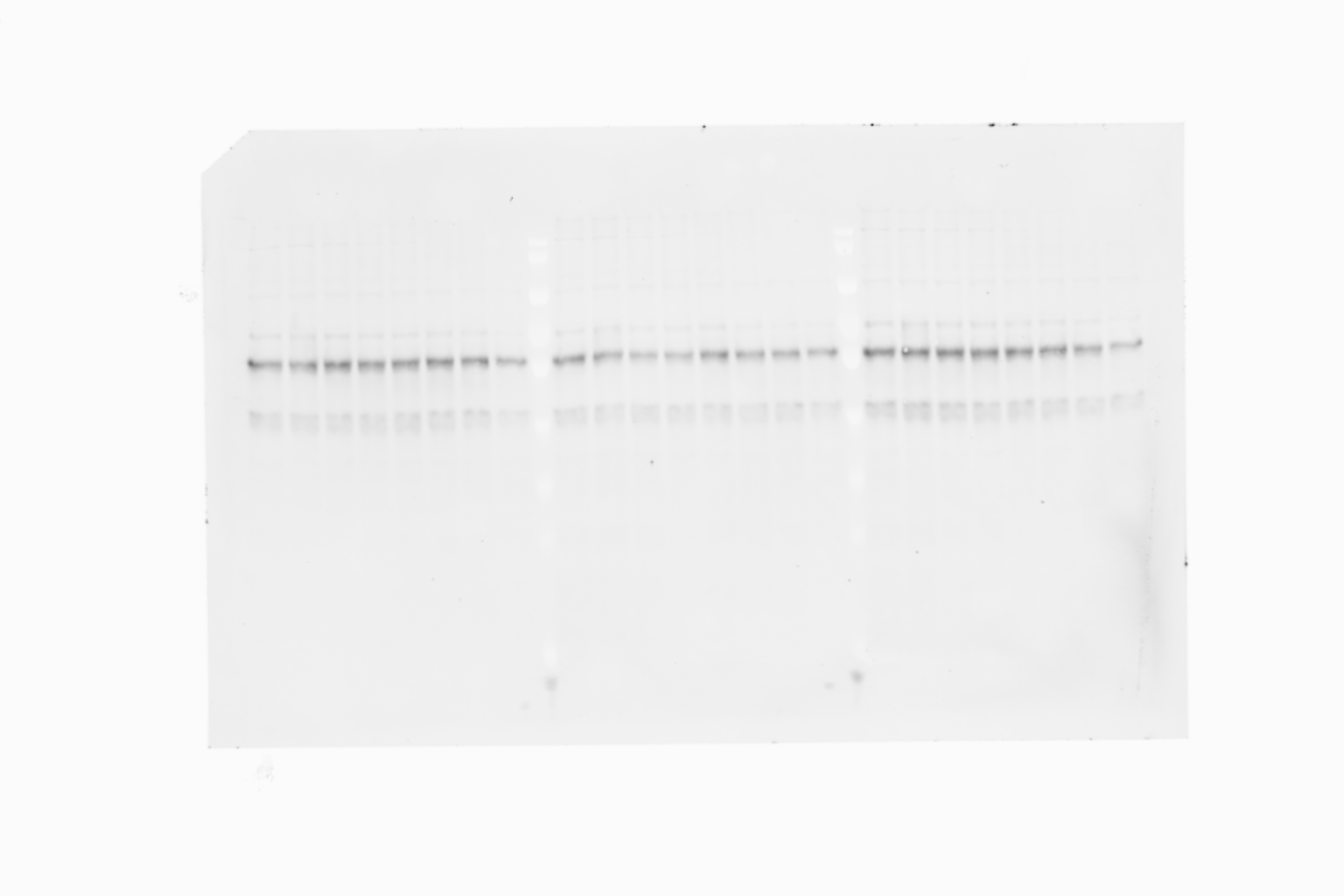

Supplement: Figure 1—source data 2. [file elife-107157-fig1-data2.zip › Figure 1A:B-source data 2/Figure 1B_anti Zwf1.tif]

A

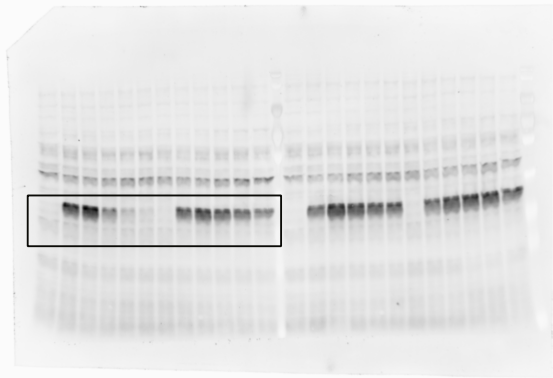

anti-YFP

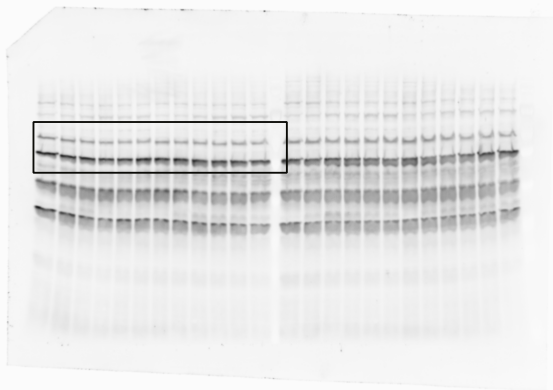

anti-Zwf1

B

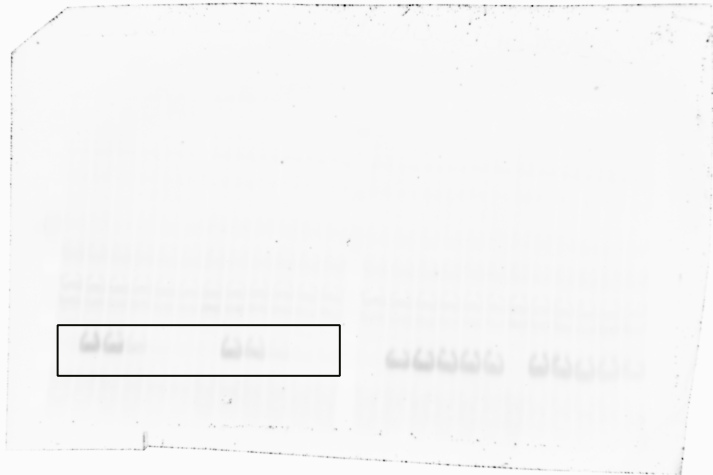

anti-YFP

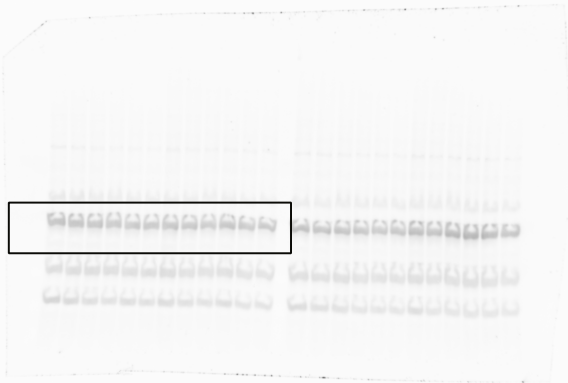

anti-Zwf1

Supplement: Figure 1—figure supplement 1—source data 1. [file elife-107157-fig1-figsupp1-data1.zip › Figure 1-figure supplement 1A:B-source data 1/Source File Figure 1 - Figure Supplement 1AB.pdf]

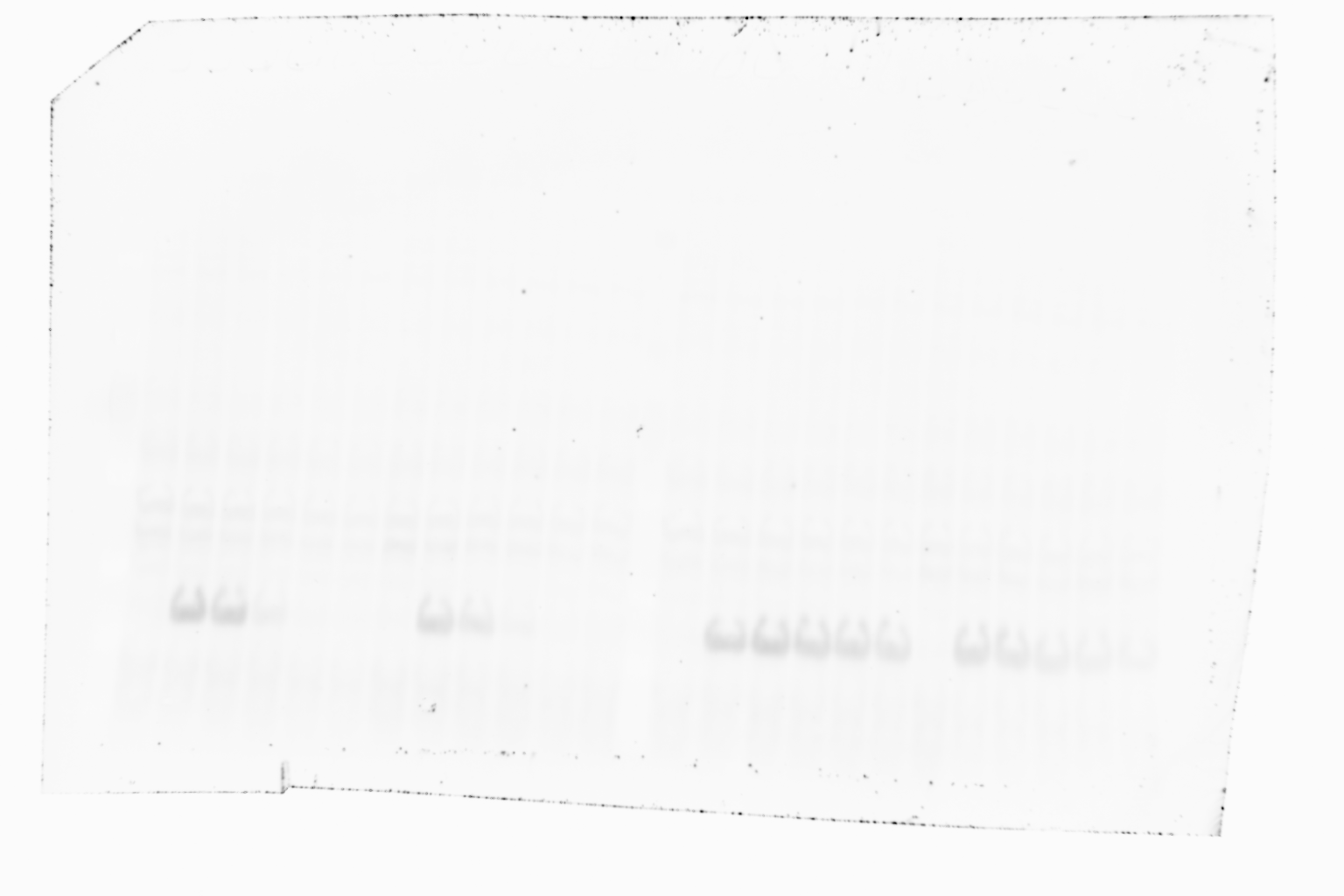

Supplement: Figure 1—figure supplement 1—source data 2. [file elife-107157-fig1-figsupp1-data2.zip › Figure 1-figure supplement 1A:B-source data 2/Figure1-figure supplement 1B_anti YFP.tif]

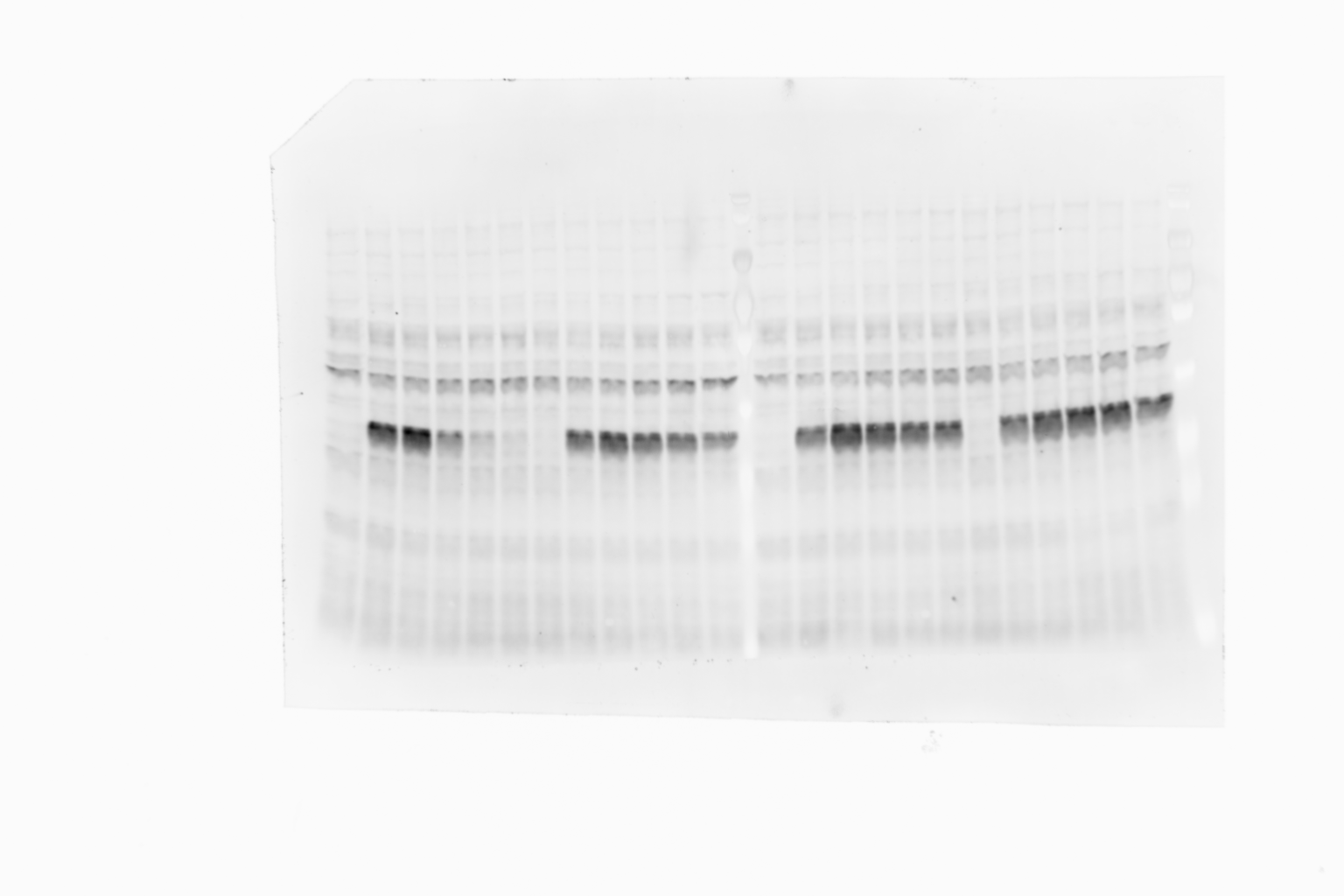

Supplement: Figure 1—figure supplement 1—source data 2. [file elife-107157-fig1-figsupp1-data2.zip › Figure 1-figure supplement 1A:B-source data 2/Figure1-figure supplement 1A_anti YFP.tif]

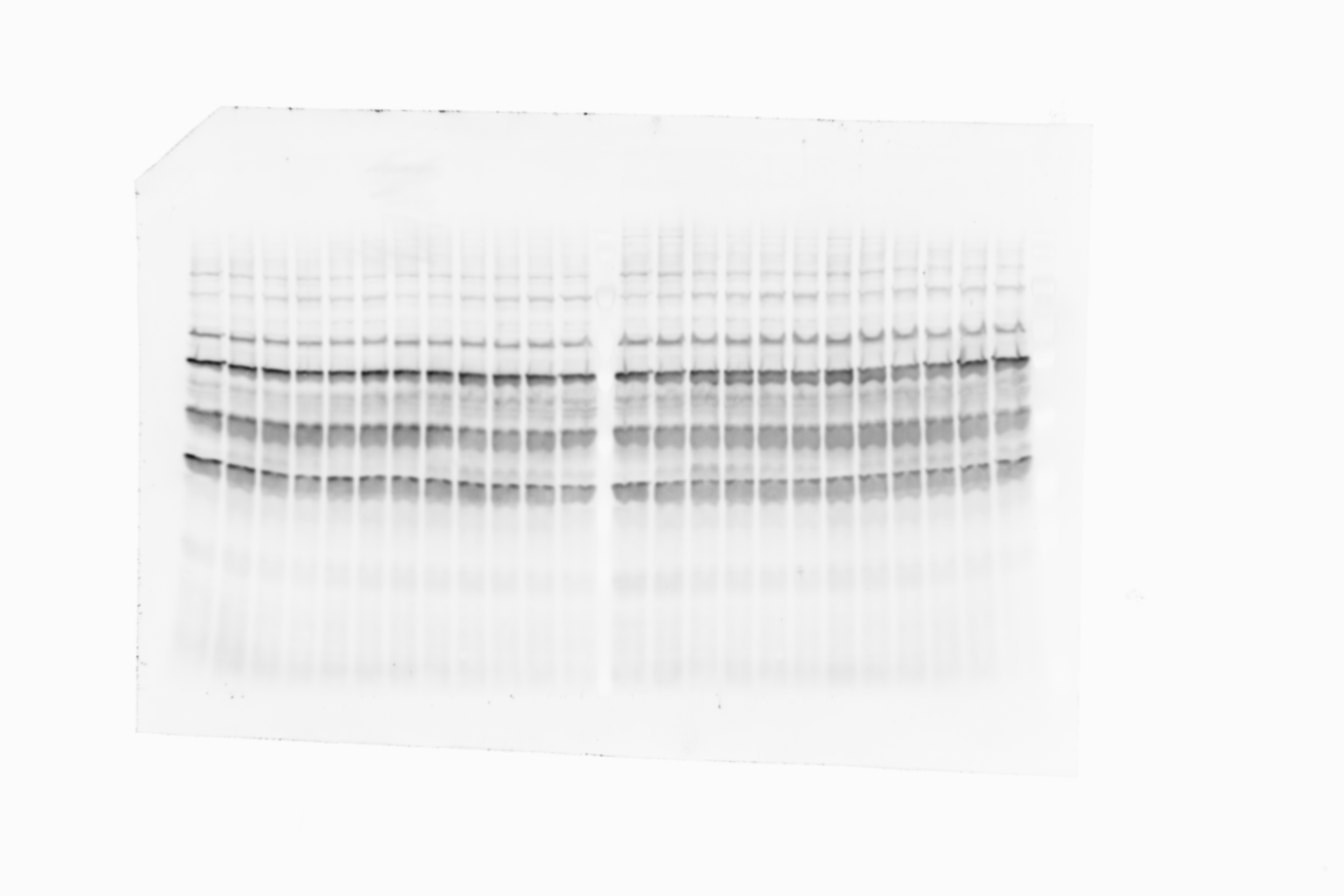

Supplement: Figure 1—figure supplement 1—source data 2. [file elife-107157-fig1-figsupp1-data2.zip › Figure 1-figure supplement 1A:B-source data 2/Figure1-figure supplement 1A_anti Zwf1.tif]

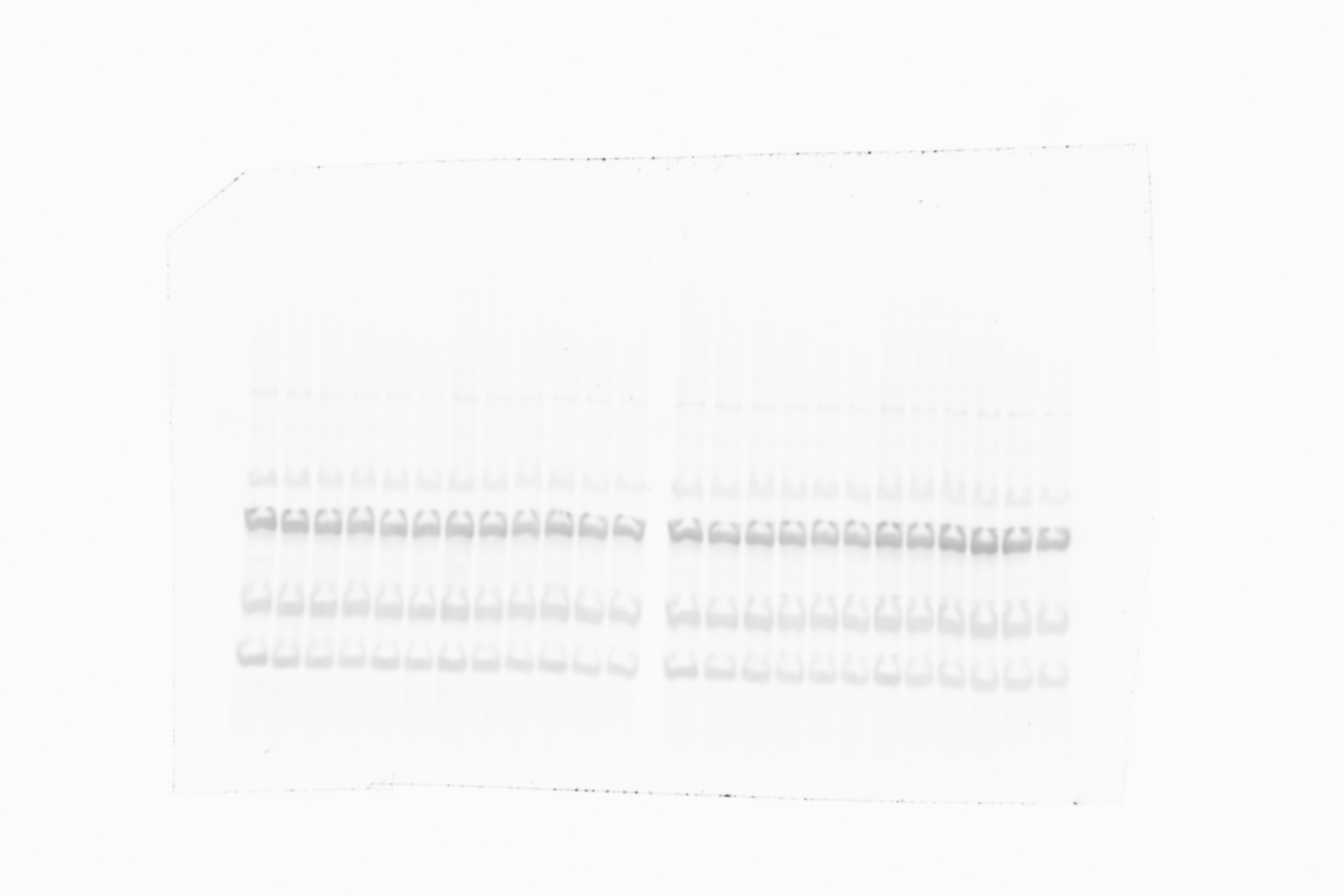

Supplement: Figure 1—figure supplement 1—source data 2. [file elife-107157-fig1-figsupp1-data2.zip › Figure 1-figure supplement 1A:B-source data 2/Figure1-figure supplement 1B_anti Zwf1.tif]

**Figure 3 - Figure Supplement 1A**

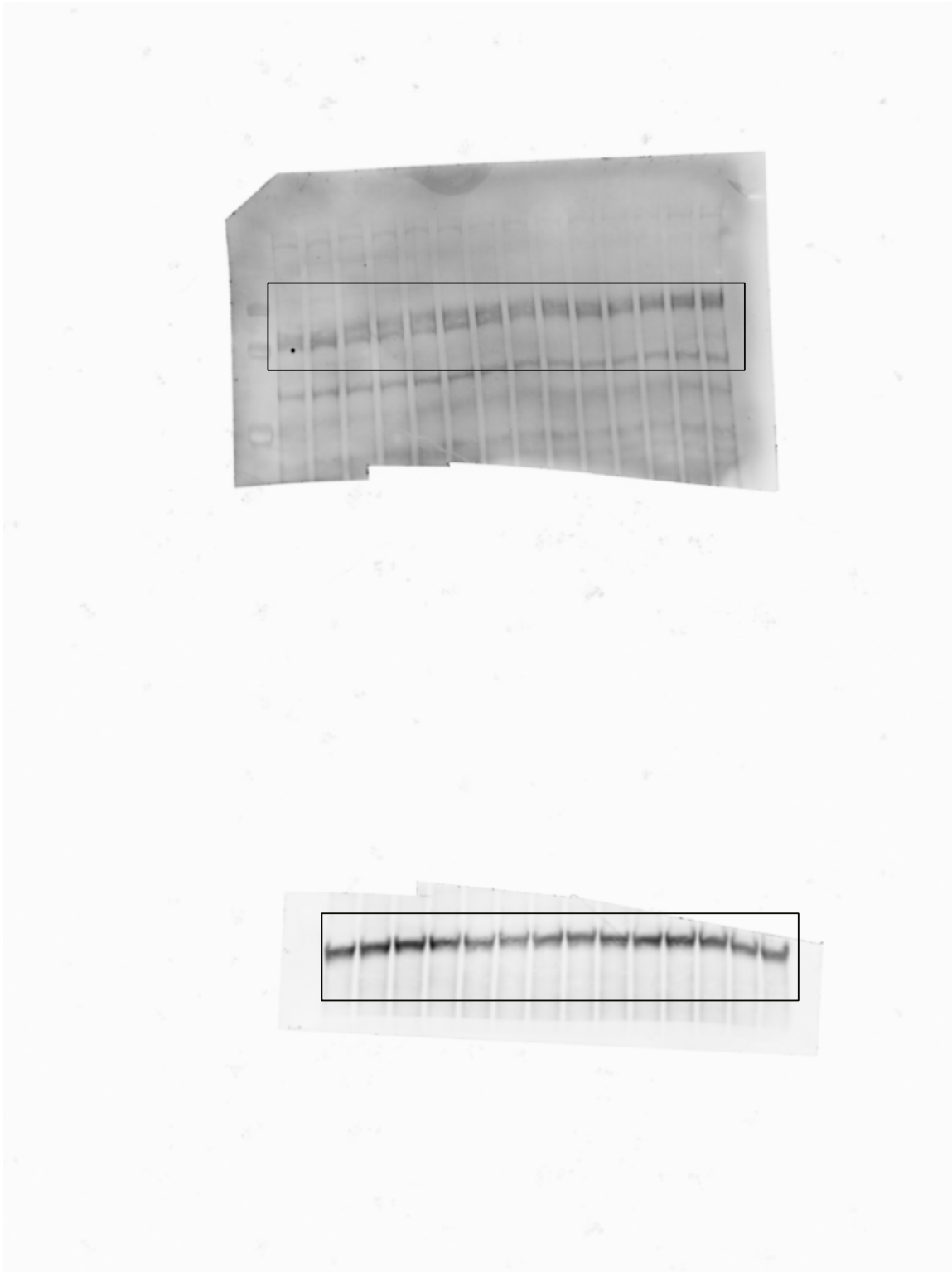

anti-V5 (Hsf1)

anti-Zwf1

Supplement: Figure 3—figure supplement 1—source data 1. [file elife-107157-fig3-figsupp1-data1.zip › Figure 3-figure supplement 1A-source data 1/Source file Figure 3 - Figure Supplement 1A.pdf]

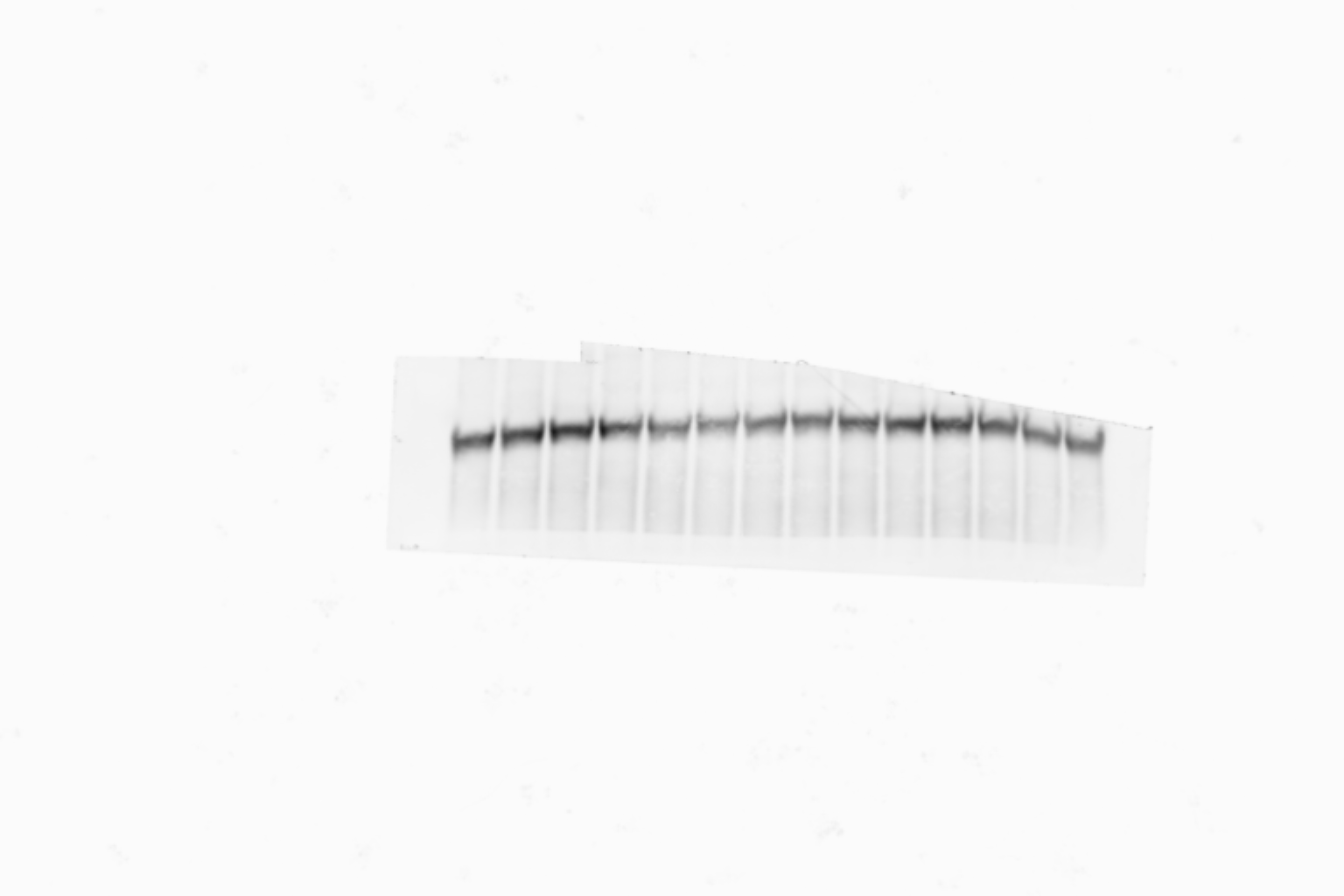

Supplement: Figure 3—figure supplement 1—source data 2. [file elife-107157-fig3-figsupp1-data2.zip › Figure 3-figure supplement 1A-source data 2/Figure 3_figure supplement 1A_anti Zwf1.tif]

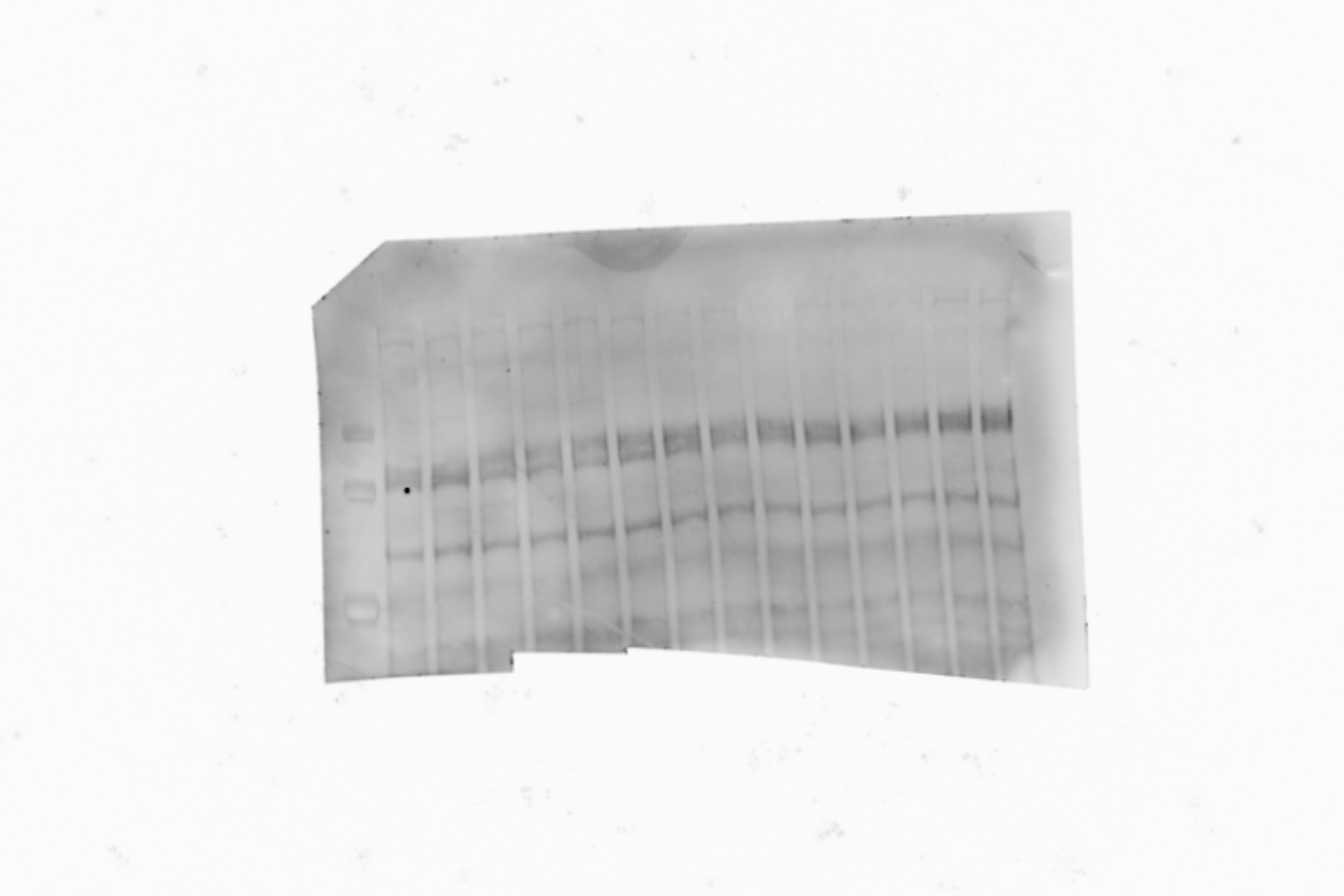

Supplement: Figure 3—figure supplement 1—source data 2. [file elife-107157-fig3-figsupp1-data2.zip › Figure 3-figure supplement 1A-source data 2/Figure 3_figure supplement 1A_anti V5.tif]

**B**

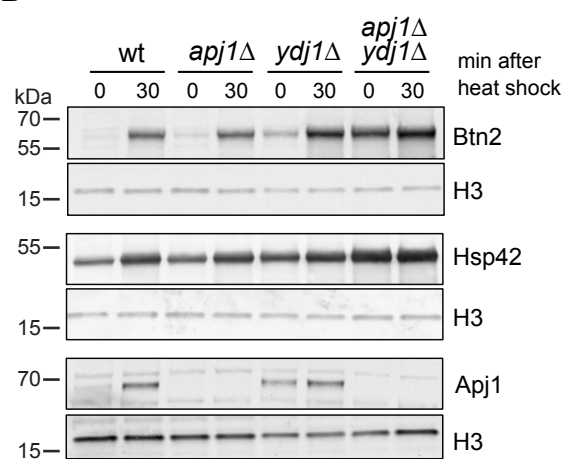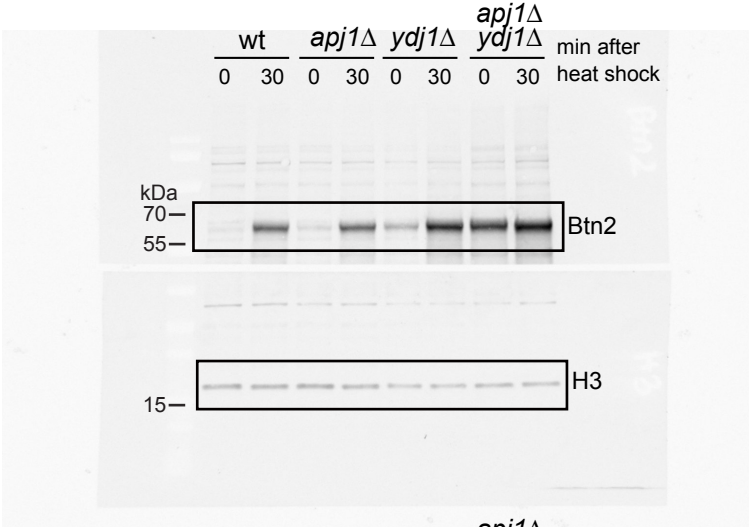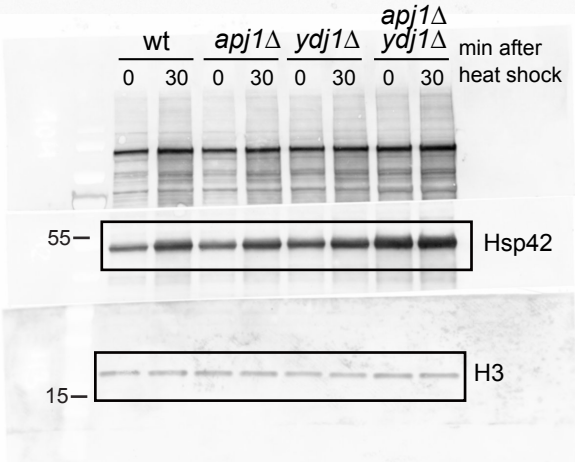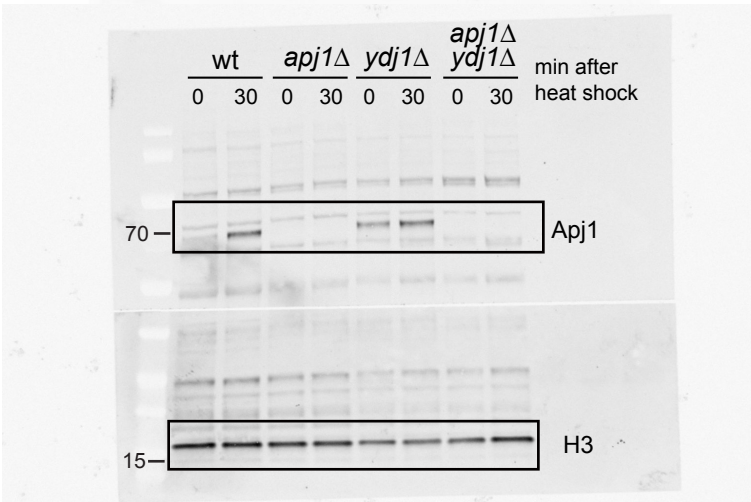

Supplement: Figure 5—source data 1. [file elife-107157-fig5-data1.zip › Figure 5B-source data 1/Source file_Figure_5B.pdf]

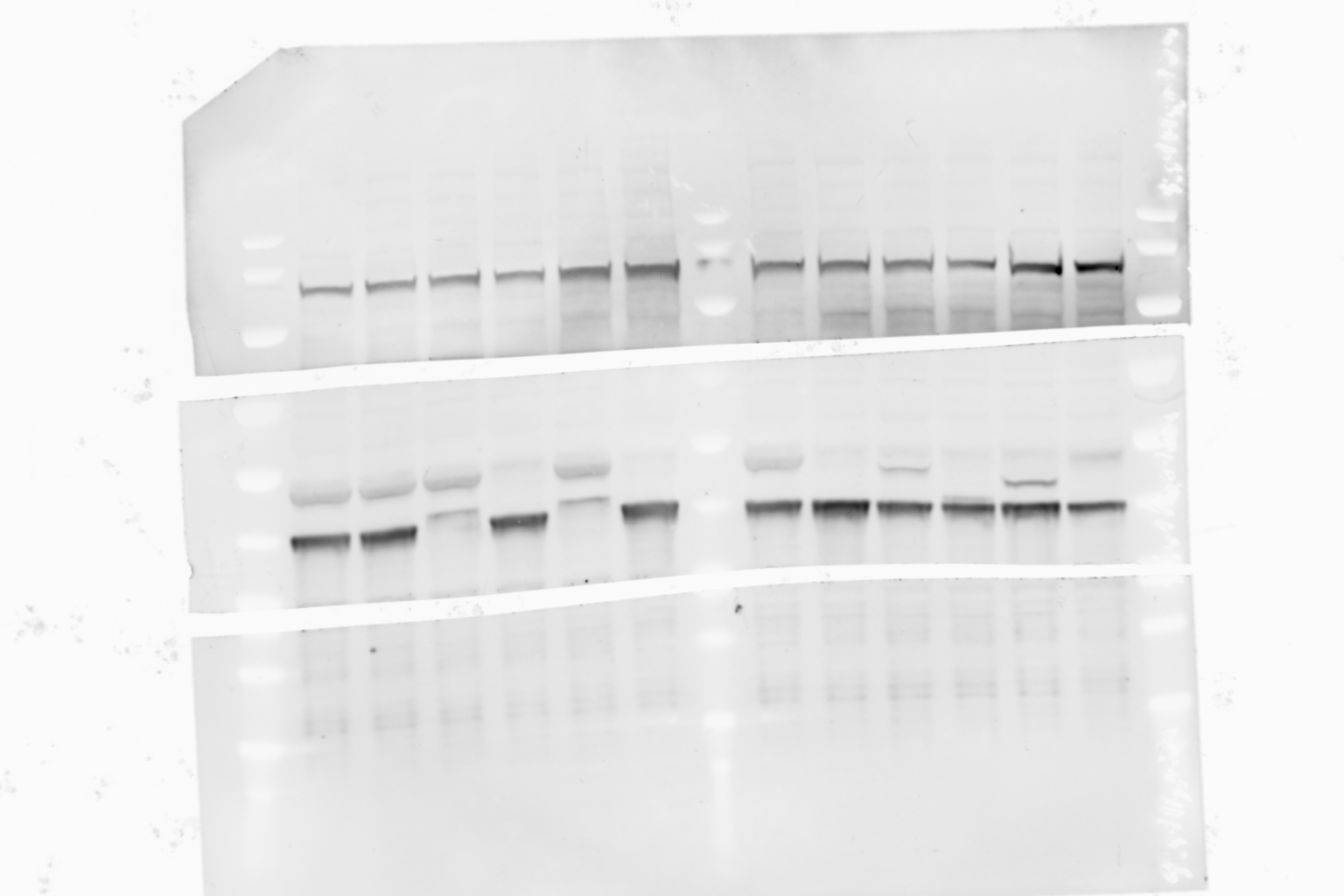

Supplement: Figure 5—figure supplement 1—source data 2. [file elife-107157-fig5-figsupp1-data2.zip › Figure 5-figure supplement 1A-source data 2/Figure5-figure supplement 1A_anti Sis1_anti H3.tif]

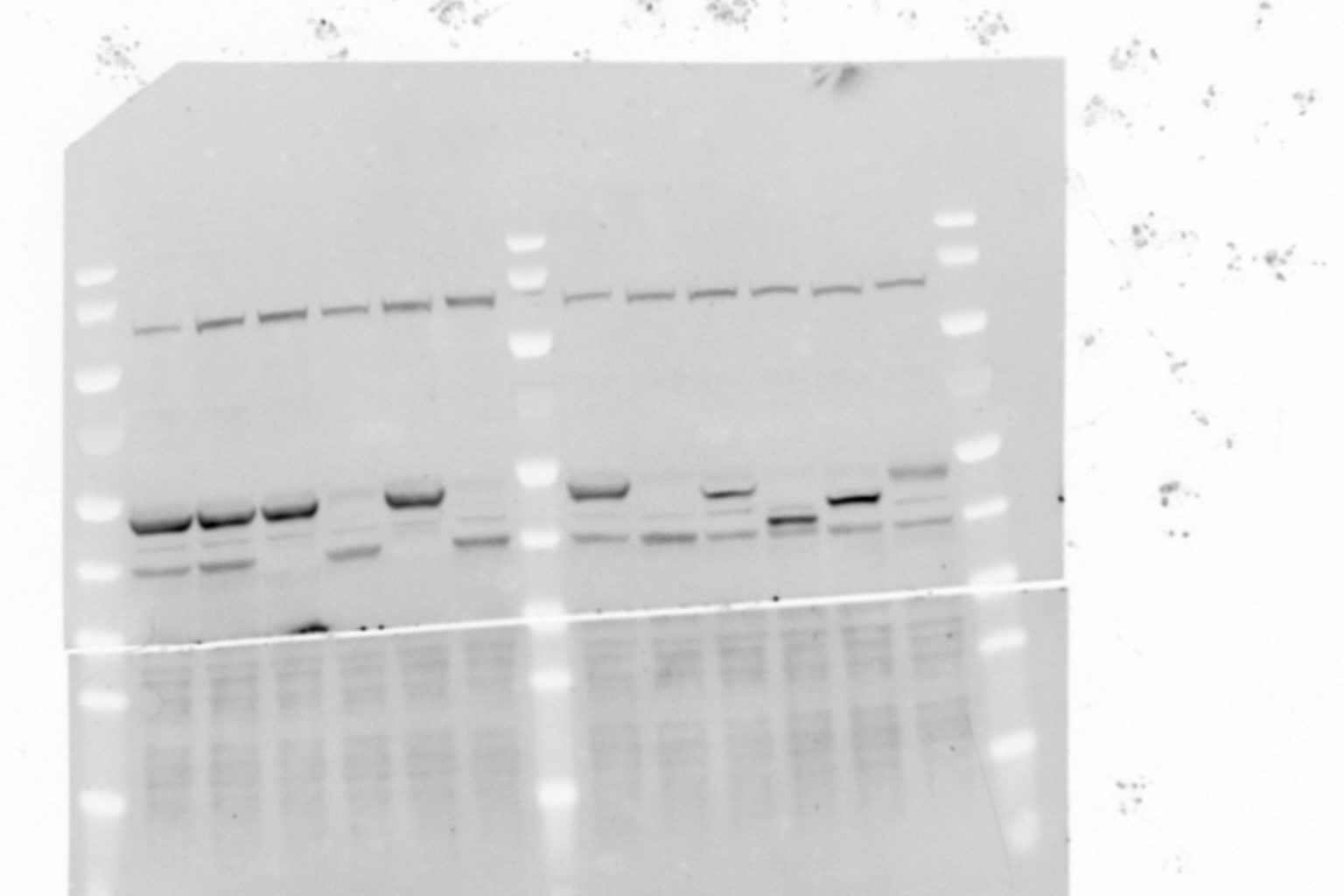

Supplement: Figure 5—figure supplement 1—source data 2. [file elife-107157-fig5-figsupp1-data2.zip › Figure 5-figure supplement 1A-source data 2/Figure5-figure supplement 1A_anti Ydj1_anti H3.tif]

**Figure 6C**

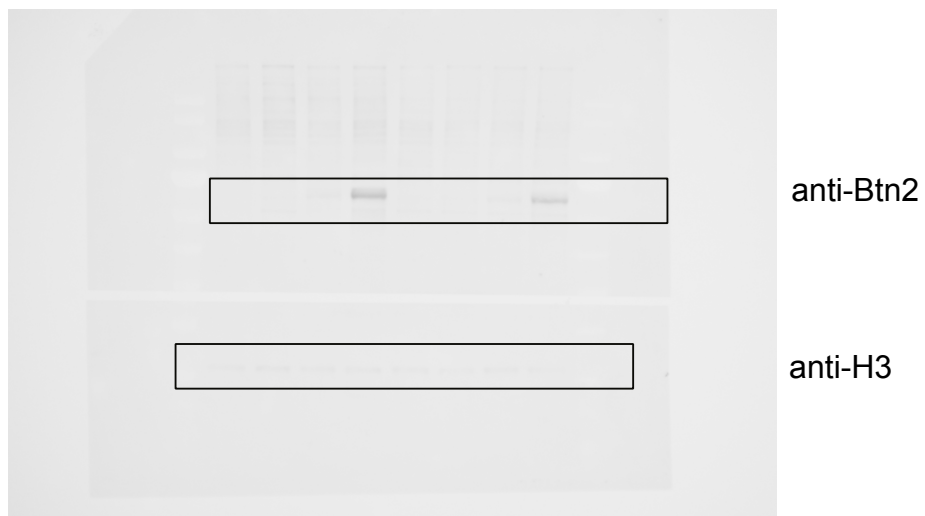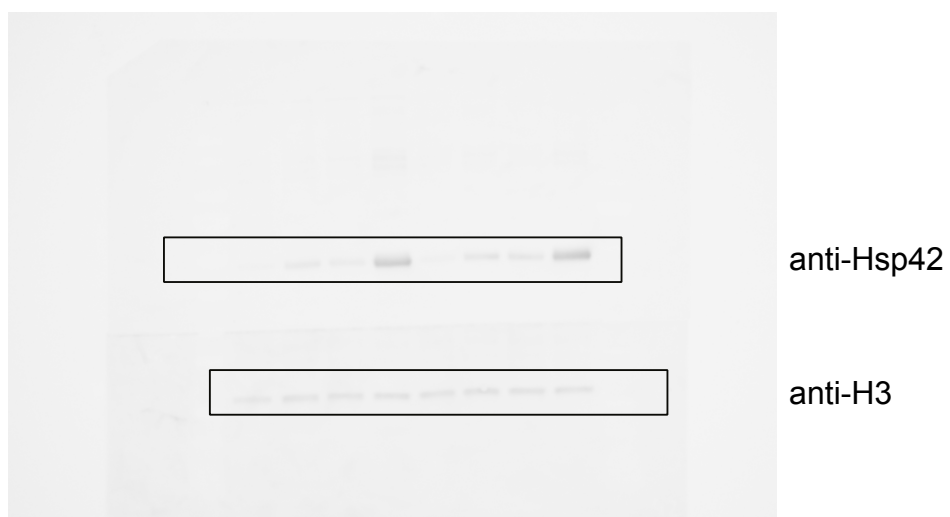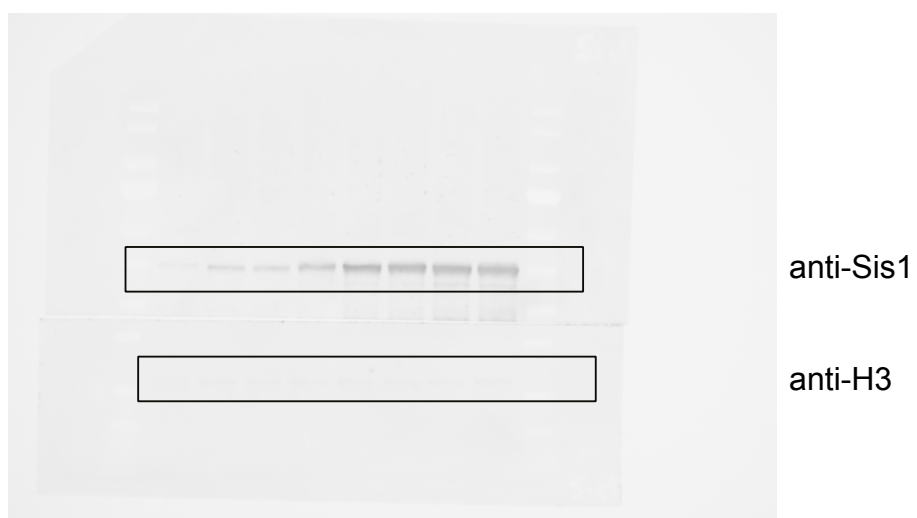

Supplement: Figure 6—source data 1. [file elife-107157-fig6-data1.zip › Figure 6C-source data 1/source File Figure6C.pdf]

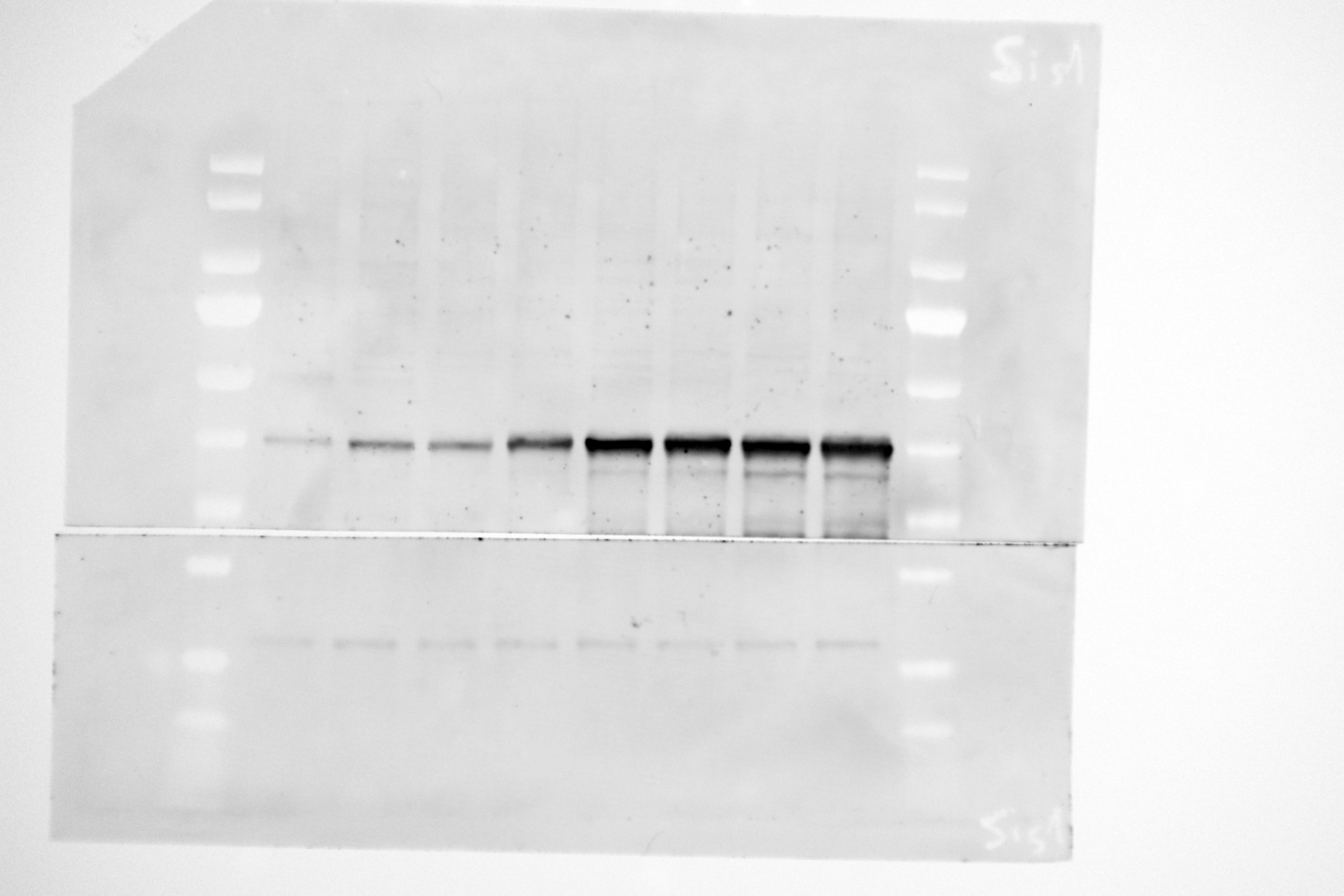

Supplement: Figure 6—source data 2. [file elife-107157-fig6-data2.zip › Figure 6C-source data 2/Figure6C_anti Sis1_anti H3.tif]

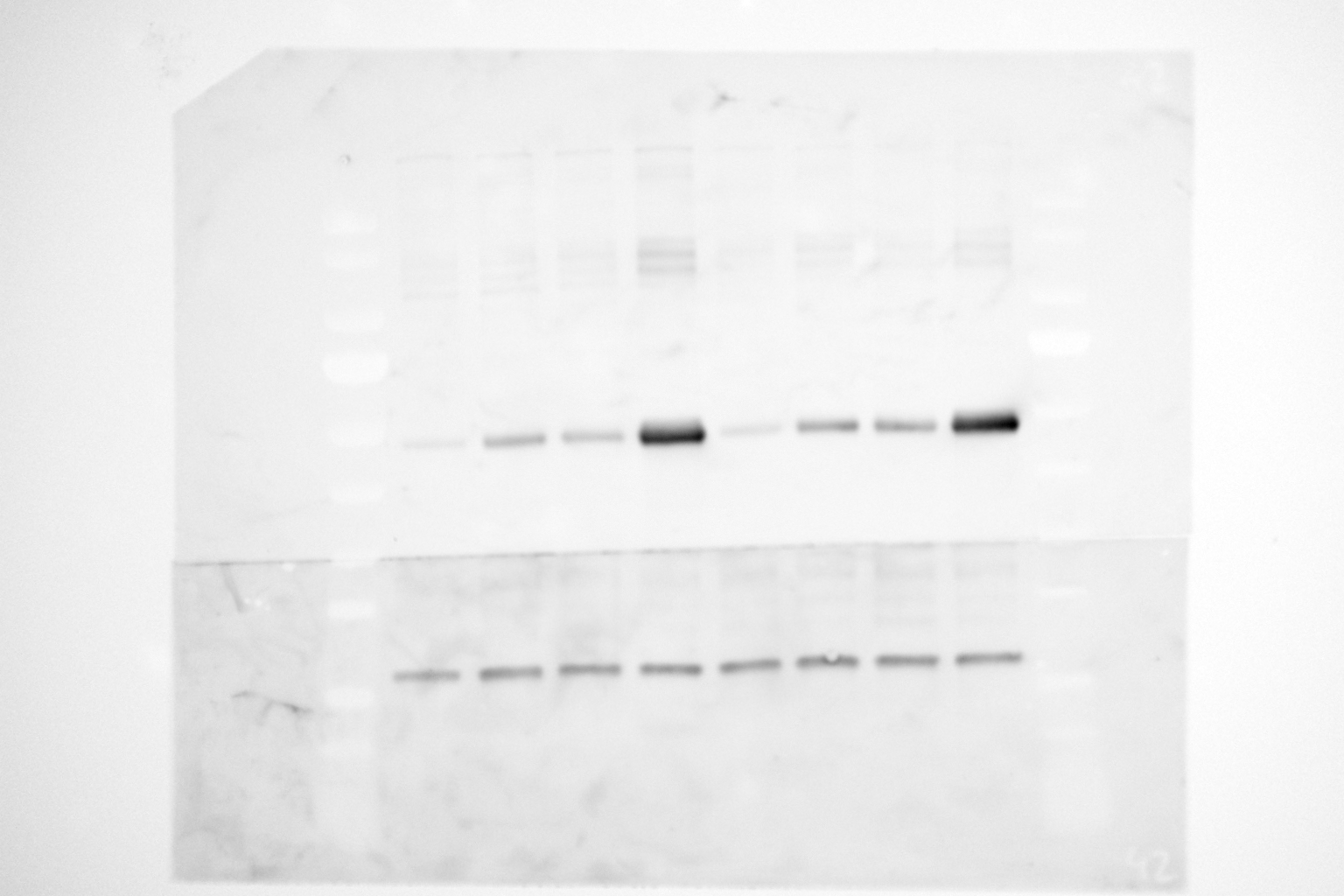

Supplement: Figure 6—source data 2. [file elife-107157-fig6-data2.zip › Figure 6C-source data 2/Figure6C_anti Hsp42_ anti H3.tif]

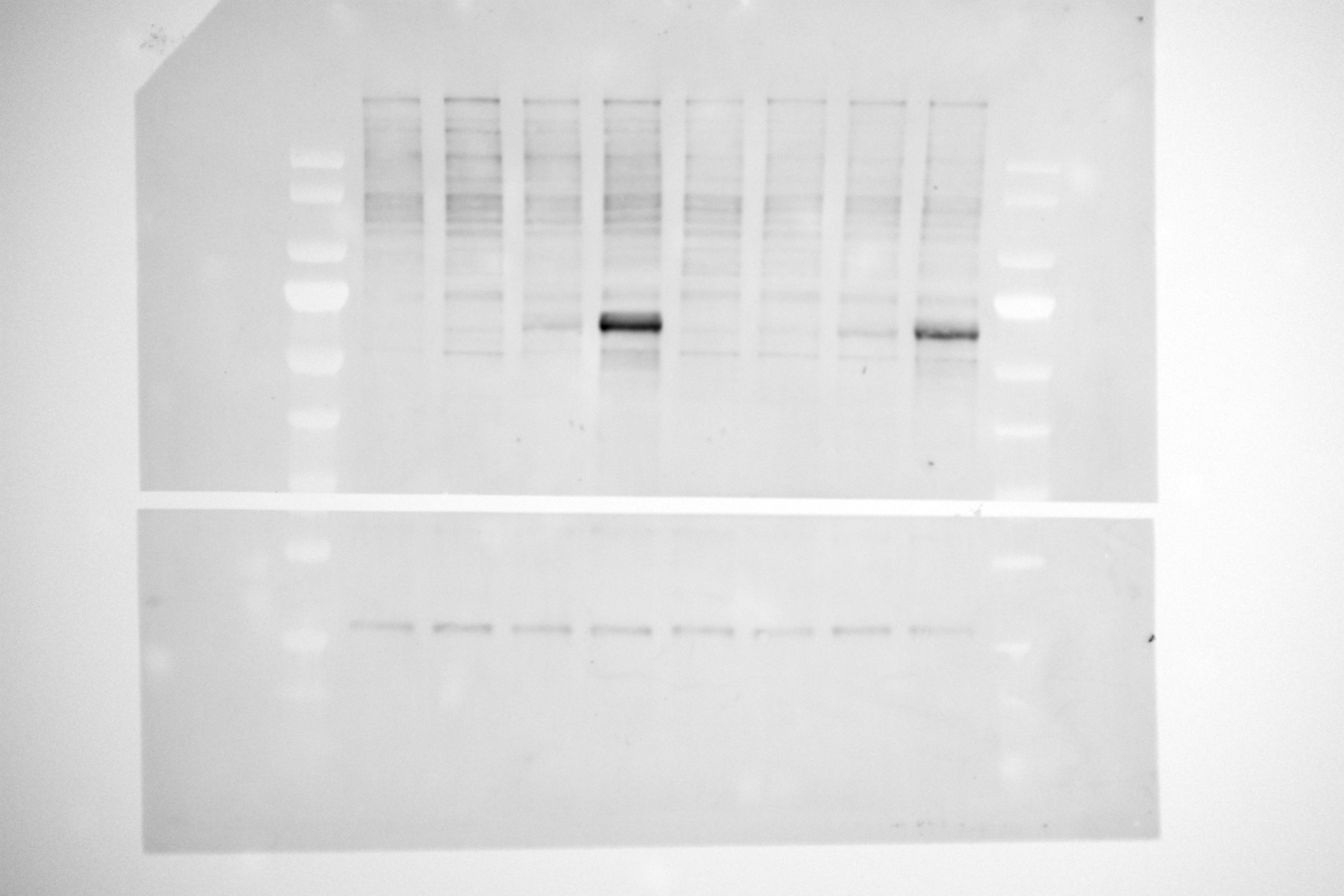

Supplement: Figure 6—source data 2. [file elife-107157-fig6-data2.zip › Figure 6C-source data 2/Figure6C_anti Btn2_anti H3.tif]

Figure 6 - Figure Supplement 1C

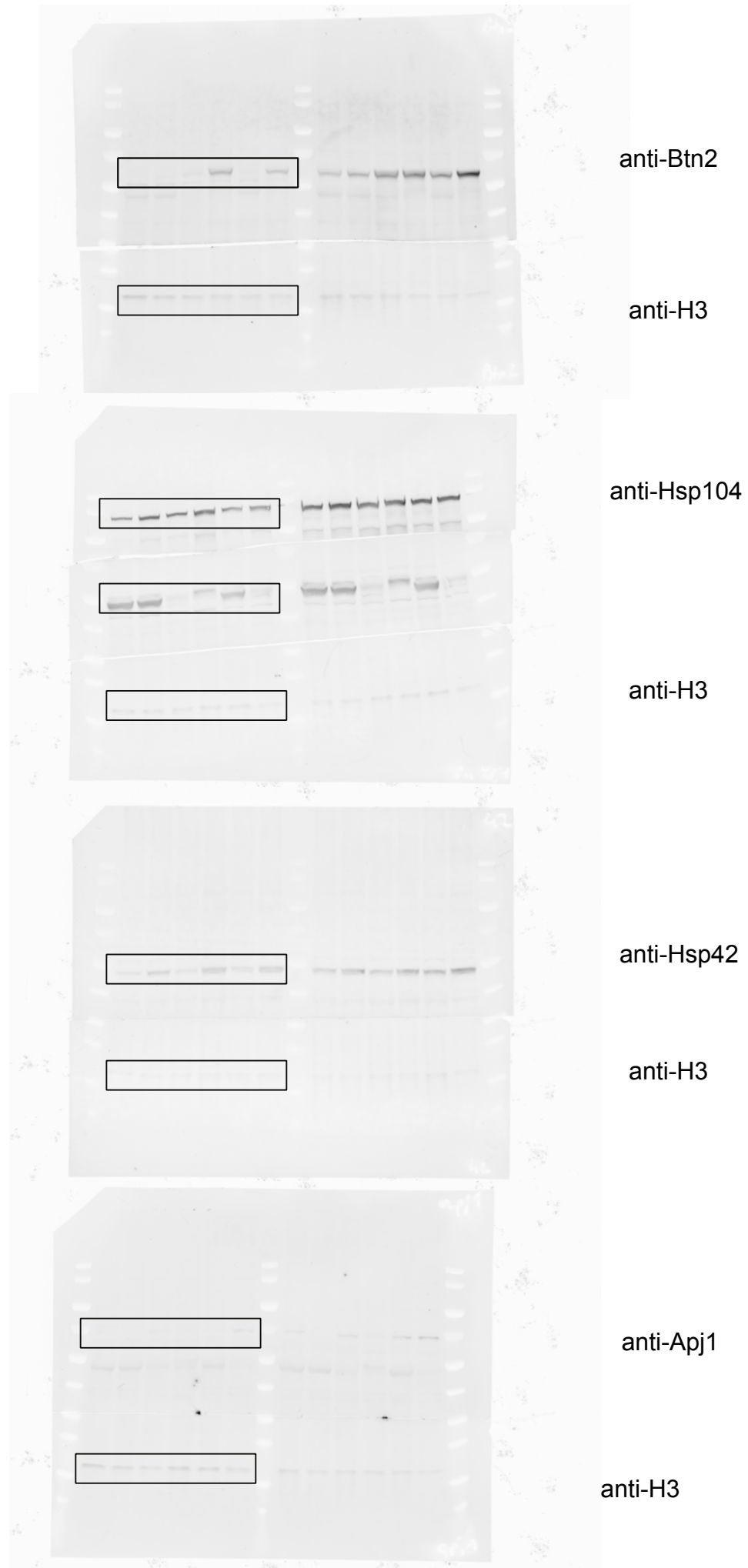

Supplement: Figure 6—figure supplement 1—source data 1. [file elife-107157-fig6-figsupp1-data1.zip › Figure 6-figure supplement 1C-source data 1/source file Figure 6 - Figure Supplement 1C.pdf]

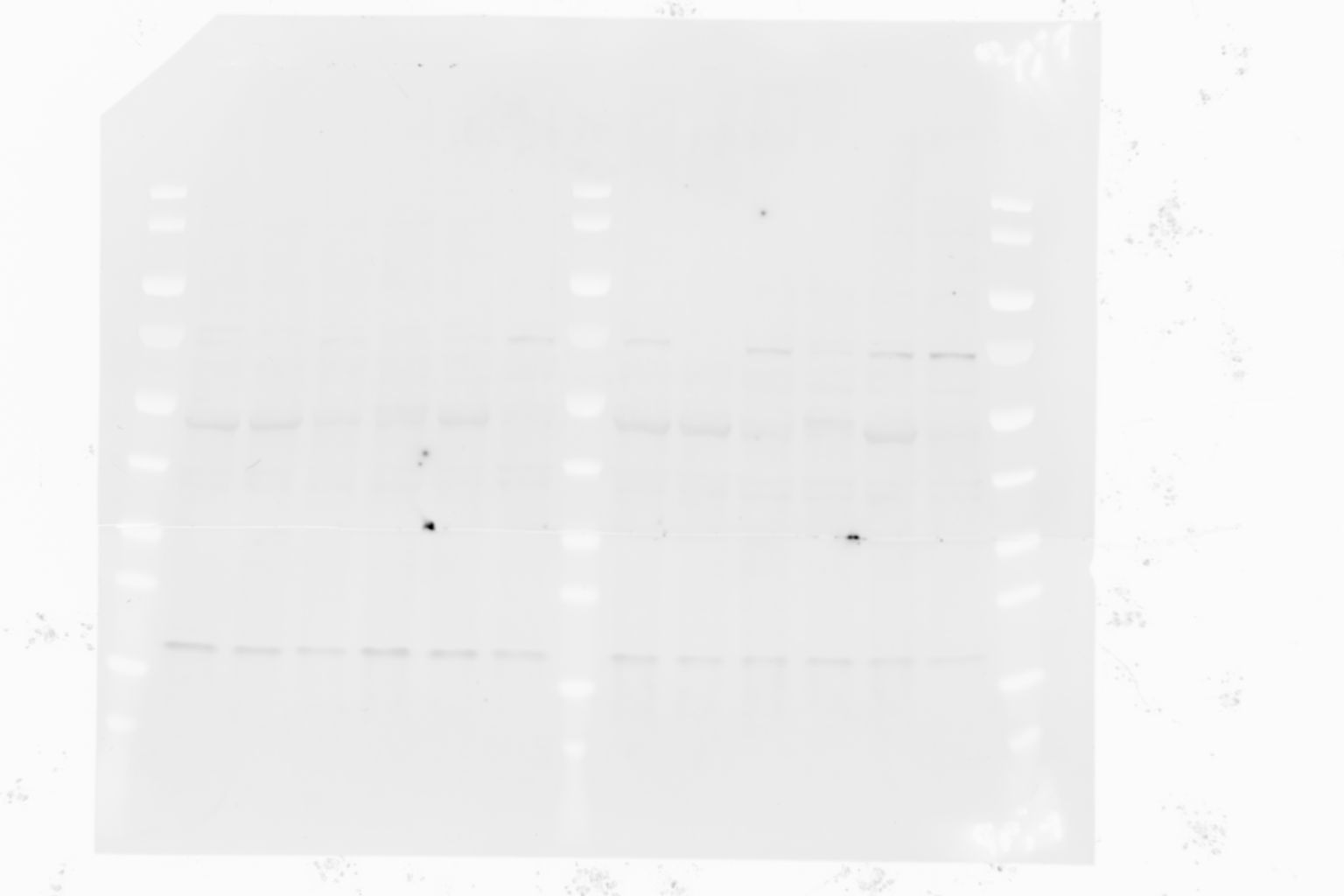

Supplement: Figure 6—figure supplement 1—source data 2. [file elife-107157-fig6-figsupp1-data2.zip › Figure 6-figure supplement 1C-source data 2/Figure6_figure supplement1C_anti Apj1_anti H3.tif]

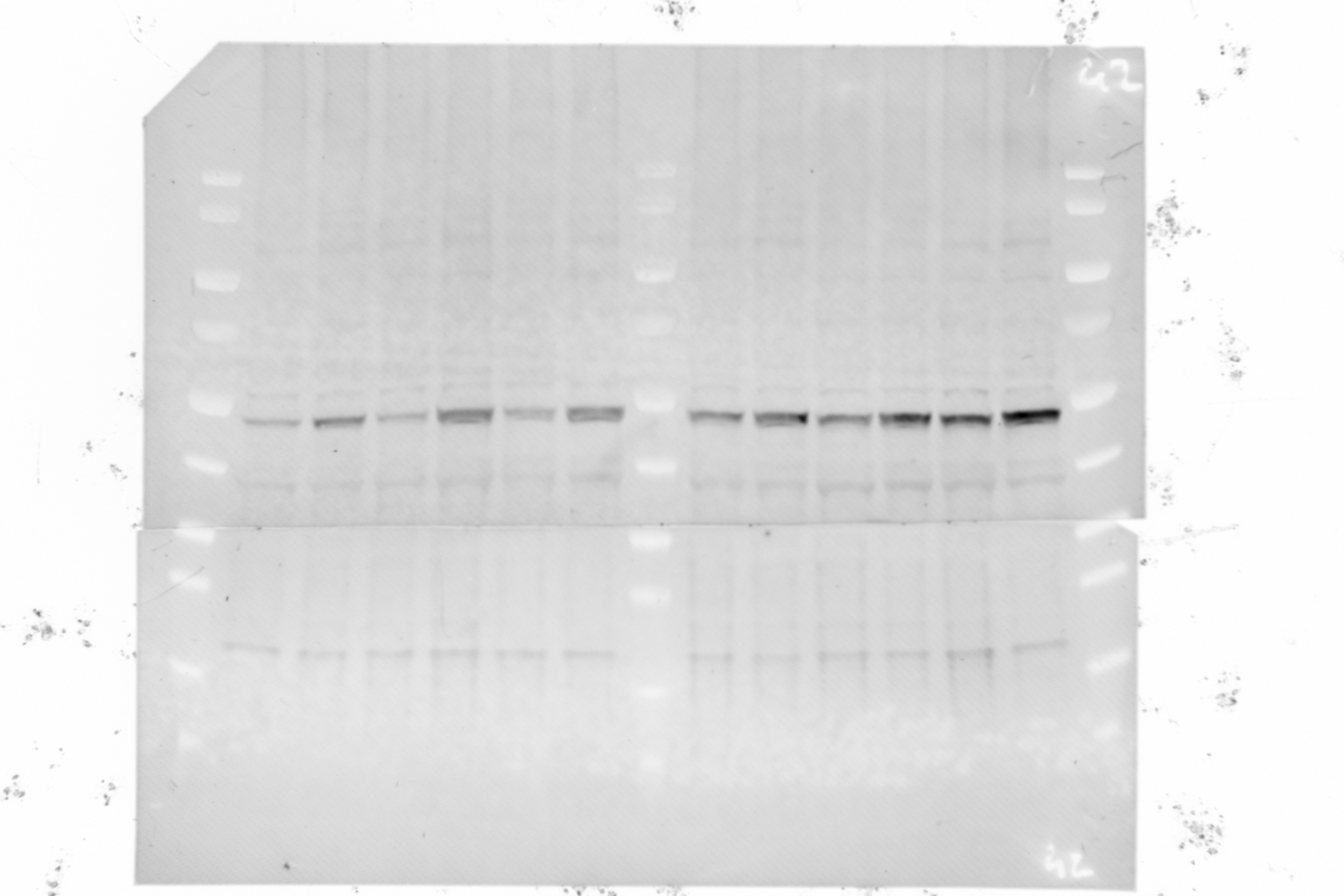

Supplement: Figure 6—figure supplement 1—source data 2. [file elife-107157-fig6-figsupp1-data2.zip › Figure 6-figure supplement 1C-source data 2/Figure6-figure supplement1C_anti Hsp42_anti H3.tif]

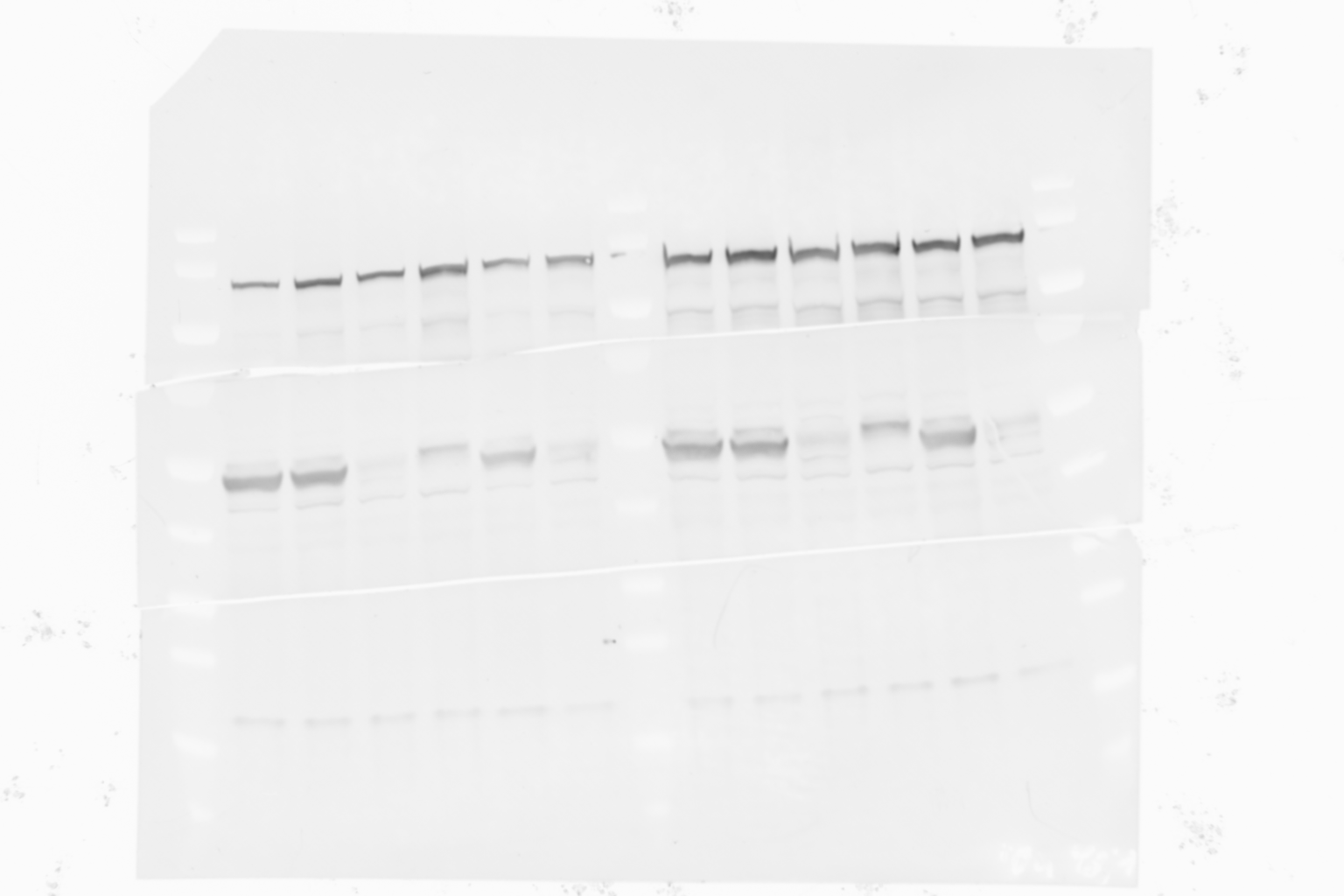

Supplement: Figure 6—figure supplement 1—source data 2. [file elife-107157-fig6-figsupp1-data2.zip › Figure 6-figure supplement 1C-source data 2/Figure6-figure supplement1C_anti Hsp104_anti H3.tif]

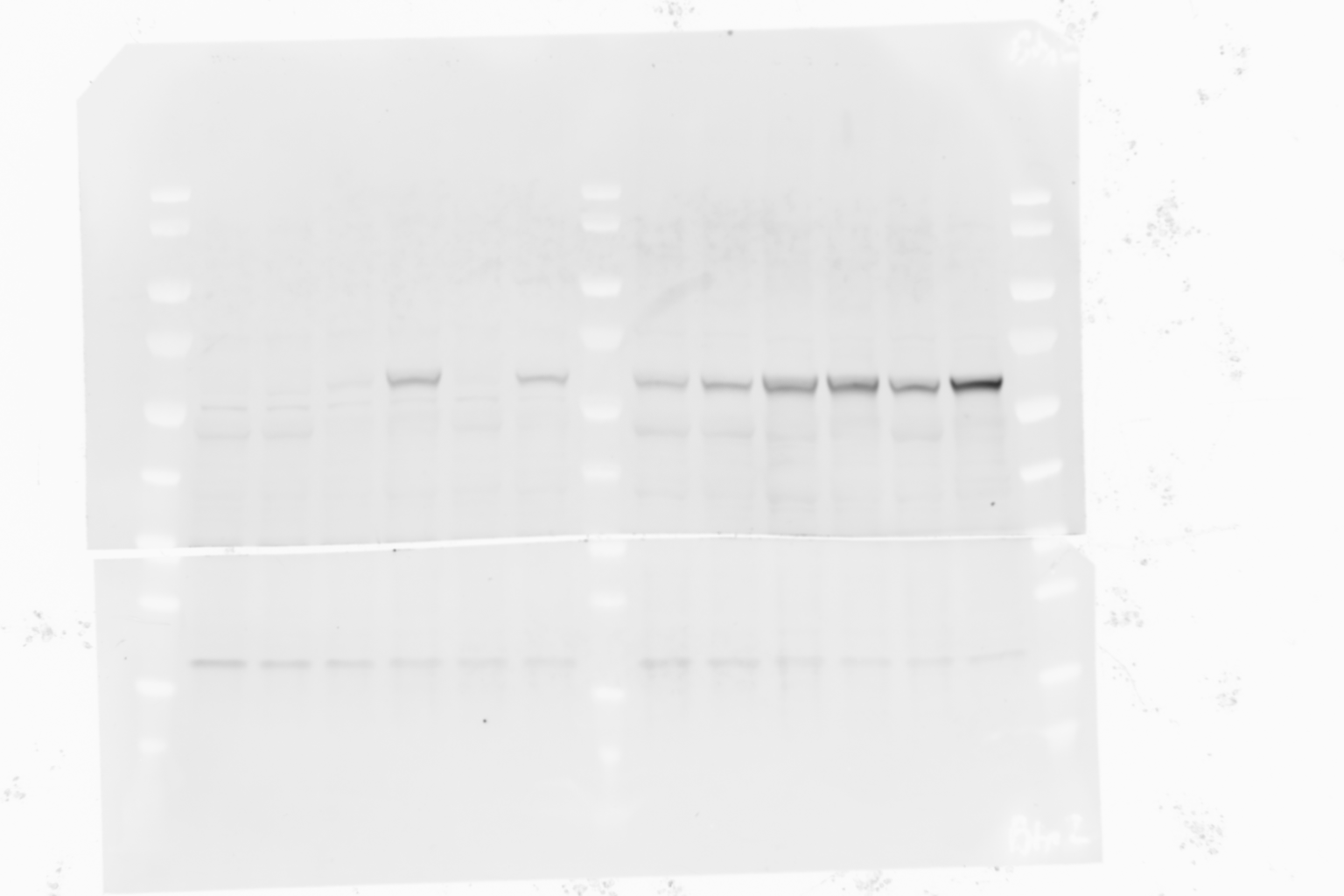

Supplement: Figure 6—figure supplement 1—source data 2. [file elife-107157-fig6-figsupp1-data2.zip › Figure 6-figure supplement 1C-source data 2/Figure6-figure supplement1C_anti Btn2_anti H3.tif]
