# Supplementary material for: Nuclear and cytosolic J-domain proteins provide synergistic control of Hsf1 at distinct phases of the heat shock response
Source: eLife. 2025 Sep 30;14:RP107157. doi: 10.7554/eLife.107157 (PMC12483511; doi:10.7554/eLife.107157)
Supplement: Supplementary file 1. [file elife-107157-supp1.docx]

Supplementary File 1: Yeast strains used in this study

| **Name** | **Fenotype** | **Genotype** | **Source** |
| --- | --- | --- | --- |
| BY4741 | wt | *MATa his3Δ1 leu2Δ0 met15Δ0 ura3Δ0* | EUROSCARF |
| W303 - 1B | wt | *MAT*α *leu2-3,112 trp1-1 can1-100 ura3-1 ade2-1 his3-11,15* | EUROSCARF |
| ASMY09 | wt | BY4741 *pdr5∆::natNT2* | this study |
| ASMY118 | wt | BY4741 *pdr5∆::natNT2*, *BTN2*prom-yeGFP_Btn2NTD*::hphNT1::HIS3* | this study |
| ASMY121 | *apj1*∆ | BY4741 *pdr5∆::natNT2*, *BTN2*prom-yeGFP_Btn2NTD*::hphNT1::HIS3*, *apj1∆::KanMX4* | this study |
| ASMY144 | Hsf1-3xFLAG-V5 | BY4741 *pdr5∆::natNT*2, *hsf1∆::KanMX4, Hsf1pr-HSF1-3xFLAG-V5::HIS3* *(C. glabarata)* | this study |
| ASMY156 | Hsf1-3xFLAG-V5, *apj1∆* | BY4741 *pdr5∆::natNT*2, *hsf1∆::KanMX4, Hsf1pr-HSF1-3xFLAG-V5::HIS3 (C. glabarata)*, *apj1∆::hphNT1* | this study |
| FA2216 | *hsf1-848* | BY4741 *hsf1-848::KanMX6,* *cir°* | this study |
| FA2220 | *hsf1-848, apj1*∆ | BY4741 *hsf1-848::KanMX6, apj1∆::hphNT1, cir°* | this study |
| CRY063 | *apj1∆* | BY4741, *apj1∆::HIS3MX6* | this study |
| CRY066 | Hsf1-GFP | BY4741 Hsf1-yeGFP::*HIS3MX6* | this study |
| CRY068 | Hsf1-GFP *apj1∆* | BY4741 Hsf1-yeGFP::*HIS3MX6, apj1∆::hphNT1* | this study |
| CRY108 | Hsf1-GFP *ydj1∆* | BY4741 Hsf1-yeGFP::*HIS3MX6, ydj1∆::URA3* | this study |
| CRY110 | Hsf1-GFP *apj1∆ydj1∆* | BY4741 Hsf1-yeGFP::*HIS3MX6, apj1∆::hphNT1, ydj1∆::URA3* | this study |
| CRY104 | *ydj1∆* | BY4741 *pdr5∆::natNT2, BTN2*prom-yeGFP_Btn2NTD::*hphNT1::HIS3,ydj1∆::URA3* | this study |
| CRY106 | *apj1∆ydj1∆* | BY4741 *pdr5∆::natNT2, BTN2*prom-yeGFP_Btn2NTD::*hphNT1::HIS3, apj1*∆::*KanMX4*, *ydj1∆::URA3* | this study |
| *ASK804* | BUD3-HS | BY4741 *UASHS-BUD3* | Chowdhary, S. Kainth, A. et al 2019 |
| GMY008 | Apj1-13xMyc | BY4741 Apj1-13XMyc::*KanMX* | this study |
| GMY011 | Apj1-13xMyc, BUD3-HS | BY4741 UASHS-BUD3, Apj1-13xMyc:*:KanMX* | this study |
| GMY019 | *apj1∆* | BY4741 *apj1∆::hphMX* | this study |
| YBK26 | *apj1∆* | BY4741, *apj1Δ::hphMX,* *pdr5Δ::natMX* | this study |
| GMY062 | Sis1-13xMyc | BY4741 Sis1-13XMyc::*KanMX* | this study |
| GMY063 | Sis1-13xMyc, *apj1∆* | BY4741 Sis1-13XMyc::*KanMX*, *apj1∆::hphMX* | this study |
| GMY064 | Ydj1-13xMyc | BY4741 Ydj1-13XMyc::*KanMX* | this study |
| GMY065 | Ydj1-13xMyc, *apj1∆* | BY4741 Ydj1-13XMyc::*KanMX*, *apj1∆::hphMX* | this study |
| SMY343 | Sis1-GFP | BY4741 pdr5Δ::natMX4, Sis1-yeGFP::*HIS3MX6* | Bukau's lab collection |
| CRY032 | Sis1-GFP, *apj1∆* | BY4741 pdr5Δ::natMX4, Sis1-yeGFP::*HIS3MX6, apj1∆::hphNT1* | this study |
| CRY170 | Sis1-GFP, *ypj1∆* | BY4741 pdr5Δ::natMX4, Sis1-yeGFP::*HIS3MX6, ydj1∆::URA3* | this study |
| CRY172 | Sis1-GFP, *apj1∆ydj1∆* | BY4741 pdr5Δ::natMX4, Sis1-yeGFP::*HIS3MX6, apj1∆::hphNT1, ydj1∆::URA3* | this study |
| JTY001 | wt | W303, GFP-lacI::*HIS3, HSP12-lacO128::URA3, HSP104-lacO256::TRP1,* *SEC63-13xMyc::KanMX,* POM34-mCherry::*natMX* | Chowdhary, S. Kainth, A. et al 2019 |
| CRY088 | *apj1∆* | W303, GFP-lacI::*HIS3, HSP12-lacO128::URA3, HSP104-lacO256::TRP1,* *SEC63-13xMyc::KanMX,* POM34-mCherry::*natMX*, *apj1∆::hphNT1* | this study |
| CRY164 | *ydj1∆* | W303, GFP-lacI::*HIS3, HSP12-lacO128::URA3, HSP104-lacO256::TRP1,* *SEC63-13xMyc::KanMX,* POM34-mCherry::*natMX*, *ydj1∆::BleMX6* | this study |
| CRY166 | *apj1∆ydj1* | W303, GFP-lacI::*HIS3, HSP12-lacO128::URA3, HSP104-lacO256::TRP1,* *SEC63-13xMyc::KanMX,* POM34-mCherry::*natMX*, *apj1∆*::hphNT1, *ydj1∆::BleMX6* | this study |
| LSY009 | *ydj1-4xcga* | BY4741, ydj1-4xcga::NatMX | this study |
| LSY012 | *sis1-4xcga* | BY4741, sis1-4xcga::NatMX | this study |
| LSY020 | *apj1∆ ydj1-4xcga* | BY4741, apj1∆::HIS3MX6, ydj1-4xcga::NatNT2 | this study |
| LSY019 | *apj1∆ sis1-4xcga* | BY4741, apj1∆::HIS3MX6, sis1-4xcga::NatNT1 | this study |
| LSY072 | *hsf1-848 ydj1-4xcga* | BY4741 hsf1-848::KanMX6, cir°, ydj1-4xcga::NatNT2 | this study |
| LSY073 | *hsf1-848 apj1∆ ydj1-4xcga* | BY4741 hsf1-848::KanMX6, apj1∆::hphNT1, cir°, ydj1-4xcga::NatNT2 | this study |
| LSY49 | *apj1H34Q* | BY4741, *apj1H34Q::HIS3MX6* | this study |
| LSY067 | *apj1H34Q ydj1-4xcga* | BY4741, apj1H34Q::HIS3MX6, ydj1-4xcga::NatNT2 | this study |
| LSY074 | wt pRS315 EV | BY4741, pRS315 | this study |
| LSY075 | *apj1∆* pRS315 EV | BY4741, *apj1∆::HIS3MX6*, pRS315 | this study |
| LSY076 | *ydj1-4xcga* pRS315 EV | BY4741, ydj1-4xcga::NatMX, pRS315 | this study |
| LSY077 | *apj1∆ ydj1-4xcga pRS315 EV* | BY4741, apj1∆::HIS3MX6, ydj1-4xcga::NatNT2, pRS315 | this study |
| LSY078 | wt TDH3:Sis1 | BY4741 pRS315 TDH3:Sis1 | this study |
| LSY079 | *apj1∆* TDH3:Sis1 | BY4741, *apj1∆::HIS3MX6*, pRS315 TDH3:Sis1 | this study |
| LSY080 | *ydj1-4xcga* TDH3:Sis1 | BY4741, ydj1-4xcga::NatMX, pRS315 TDH3:Sis1 | this study |
| LSY081 | *apj1∆ ydj1-4xcga TDH3:Sis1* | BY4741, apj1∆::HIS3MX6, ydj1-4xcga::NatNT2, pRS315 TDH3:Sis1 | this study |
