# Supplementary material for: Nuclear and cytosolic J-domain proteins provide synergistic control of Hsf1 at distinct phases of the heat shock response
Source: eLife. 2025 Sep 30;14:RP107157. doi: 10.7554/eLife.107157 (PMC12483511; doi:10.7554/eLife.107157)
Supplement: Supplementary file 2. [file elife-107157-supp2.docx]

Supplementary File 2: Plasmids used in this study

| **Backbone** | **Insert** | Source |
| --- | --- | --- |
| pRS315 | empty vector | Bukau's Lab collection |
| pRS315 | TDH3:Sis1 | Brandman's lab collection |
| pFA876 | pRS315 pApj1 GFP-Apj1-AAA | this study |
| pFA711 | pCU426 pGAL GFP | this study |
