# Supplementary material for: Nuclear and cytosolic J-domain proteins provide synergistic control of Hsf1 at distinct phases of the heat shock response
Source: eLife. 2025 Sep 30;14:RP107157. doi: 10.7554/eLife.107157 (PMC12483511; doi:10.7554/eLife.107157)
Supplement: Supplementary file 3. [file elife-107157-supp3.docx]

Supplementary File 3: Antibodies used in this study

| Antibody | Dilution | Source |  |
| --- | --- | --- | --- |
| ⍺-Btn2 (rabbit) | 1:5000 | Bukau's Lab collection |  |
| ⍺-Hsp42 (rabbit) | 1:5000 | Bukau's Lab collection |  |
| ⍺-Zwf1 (rabbit) | 1:50000 | Bukau's Lab collection |  |
| ⍺-Apj1 (rabbit) | 1:2000 | den Brave's Lab collection |  |
| ⍺-FLAG (mouse) | 1:10000 | Sigma-Aldrich |  |
| ⍺-H3 (rabbit) | 1:10000 | Sigma-Aldrich |  |
| ⍺-Sis1 (rabbit) | 1:5000 | Bukau's Lab collection |  |
| ⍺-Ydj1 (rabbit) | 1:5000 | Bukau's Lab collection |  |
| ⍺-Hsp104 (rabbit) | 1:20000 | Bukau's Lab collection |  |
| ⍺-GFP (rabbit) | 1:1000 | Bukau's Lab collection |  |
| ⍺-cMyc (mouse) | 1:360 | Santa Cruz Biotechnology | 2.5 µl of Ab per sample (ChIP) |
| ⍺-Hsf1 | 1:600 | Gross' Lab collection | 1.5 µl of Ab per sampl (ChIP) |
| Alkaline Phosphatase Goat Anti-Rabbit IgG, AP1000 | 1:2500- 1:10000 | Vector Laboratories |  |
| Alkaline Phosphatase Goat Anti-Mouse IgG, AP2000 | 1:2500- 1:10000 | Vector Laboratories |  |
|  |  |  |  |
|  |  |  |  |
