# Supplementary material for: Nuclear and cytosolic J-domain proteins provide synergistic control of Hsf1 at distinct phases of the heat shock response
Source: eLife. 2025 Sep 30;14:RP107157. doi: 10.7554/eLife.107157 (PMC12483511; doi:10.7554/eLife.107157)
Supplement: Supplementary file 4. [file elife-107157-supp4.docx]

Supplementary File 4: Sequences of RT-PCR primer used in this study

| **Figure 3B** |  |
| --- | --- |
| **Primer Name** | **Sequence (5’ to 3’)** |
| pTOS1_RT_for | ACCGACTAATGCGGTCATGGAAAGC |
| pTOS1_RT_rev | CTTTTCTCGCAAGAAGACTCCAGAATCA |
| pSSA4_RT_for | GGATATCTTTTGCCCGGTGAGTTG |
| pSSA4_RT_rev | TGTCGTCAAACTAAGGAGCTTCCC |
| pUBI4_RT_for | GGAGCATCACACAGCCGTACATC |
| pUBI4_RT_rev | AAAAGGAGGAACCGCCCTCAAATG |
| pSSA3_RT_for | GATGCCTATGGAGGTTATGGGTGC |
| pSSA3_RT_rev | CCCTTCCATTCGTTTCCAATTGTGC |
| pHSP42_RT_for | CACGCGCTTAAAAGTTCTGGAAGG |
| pHSP42_RT_rev | AACTAACTTCACAGAGGCCTCCCC |
| pBTN2_RT_for | GTGGAGCTCGAGAGTTGTATCCAG |
| pBTN2_RT_rev | CGCCAAGAACTGAAGGCTTCTATG |
|  |  |
| **Figure 3D/E, Figure 4, Figure 3 - Figure Supplement 1, Figure 4 - Figure Supplements 1/2** | |
| ARS504 FP | GTC AGA CCT GTT CCT TTA AGA GG |
| ARS504 RP | CAT ACC CTC GGG TCA AAC AC |
| HSP104 UAS-267 FP | CTT AAA CGT TCC ATA AGG GGC |
| HSP104 UAS-216 RP | TGC AGT TCT TTG AGA TGG GCC |
| HSP82 UAS -394 FP | CCT CTC TCA ACA CAG TAA TCC ATA AAC |
| HSP82 UAS -242 RP | CTT CCA CGG CGT TCT AGA AAA AAA AG |
| SSA4 UAS -374 FP | GCC GCA CAT CCA TTC CGG TAT G |
| SSA4 UAS -312RP | CGG GCA AAA GAT ATC CGC TTT G |
| SSA1 UAS -428 FP | CGGTGTGTGGATGATGGTTTCATCAT |
| SSA1 UAS -178 RP | GTCCTCGAAACGATCAGCTAATCTAAATGG |
| HSP42 UAS -371 FP | GGATATGACATACTTCAATTCAGC |
| HSP42 UAS -160 RP | CAAGTCTTATATAACTAACTTCACAGAGG |
| BTN2 UAS -406 FP | GTCATGTAGCACTATTTCAGCC |
| BTN2 UAS -220 RP | CATTTGTTTTGCCACTTTACTTCG |
